# Identification of clade-wide putative *cis*-regulatory elements from conserved non-coding sequences in Cucurbitaceae genomes

**DOI:** 10.1093/hr/uhad038

**Published:** 2023-02-28

**Authors:** Hongtao Song, Qi Wang, Zhonghua Zhang, Kui Lin, Erli Pang

**Affiliations:** MOE Key Laboratory for Biodiversity Science and Ecological Engineering and Beijing Key Laboratory of Gene Resource and Molecular Development, College of Life Sciences, Beijing Normal University, Beijing 100875, China; MOE Key Laboratory for Biodiversity Science and Ecological Engineering and Beijing Key Laboratory of Gene Resource and Molecular Development, College of Life Sciences, Beijing Normal University, Beijing 100875, China; College of Horticulture, Qingdao Agricultural University, Qingdao 266109, China; MOE Key Laboratory for Biodiversity Science and Ecological Engineering and Beijing Key Laboratory of Gene Resource and Molecular Development, College of Life Sciences, Beijing Normal University, Beijing 100875, China; MOE Key Laboratory for Biodiversity Science and Ecological Engineering and Beijing Key Laboratory of Gene Resource and Molecular Development, College of Life Sciences, Beijing Normal University, Beijing 100875, China

## Abstract

*Cis*-regulatory elements regulate gene expression and play an essential role in the development and physiology of organisms. Many conserved non-coding sequences (CNSs) function as *cis*-regulatory elements. They control the development of various lineages. However, predicting clade-wide *cis*-regulatory elements across several closely related species remains challenging. Based on the relationship between CNSs and *cis*-regulatory elements, we present a computational approach that predicts the clade-wide putative *cis*-regulatory elements in 12 Cucurbitaceae genomes. Using 12-way whole-genome alignment, we first obtained 632 112 CNSs in Cucurbitaceae. Next, we identified 16 552 Cucurbitaceae-wide *cis*-regulatory elements based on collinearity among all 12 Cucurbitaceae plants. Furthermore, we predicted 3 271 potential regulatory pairs in the cucumber genome, of which 98 were verified using integrative RNA sequencing and ChIP sequencing datasets from samples collected during various fruit development stages. The CNSs, Cucurbitaceae-wide *cis*-regulatory elements, and their target genes are accessible at http://cmb.bnu.edu.cn/cisRCNEs_cucurbit/. These elements are valuable resources for functionally annotating CNSs and their regulatory roles in Cucurbitaceae genomes.

## Introduction

Conserved non-coding sequences (CNSs) display an extraordinary degree of conservation between two or more species. They often cluster around genes that act as *cis*-regulatory elements in development and differentiation [1] and have recently emerged as novel phylogenomic markers [2].

CNSs are defined by different names in literature, such as CNEs (conserved noncoding elements [1]) or UCEs (ultraconserved noncoding elements [3]). CNSs were first discovered by comparing the human, rat, fly, yeast, and mouse genomes [3–5]. Recently, plant CNSs have been identified on a genome-wide scale by comparing one model species with several related genomes. Zheng *et al.* [6] first identified 25 ultra-conserved elements longer than 100 bp by comparing the *Arabidopsis thaliana* and rice genomes. Kritsas *et al.* [7] found the properties of plant CNSs, such as strong purifying selection, sharp decline in A + T content at the CNS border, and enrichment of genes encoding transcription factors and genes involved in development. Whole-genome alignment of 20 flowering plants detected a large set of previously uncharacterized intergenic elements [8]. Haudry *et al.* [9] surveyed the nine-way alignment conservation of crucifer genomes and identified approximately 90 000 crucifer CNSs potentially involved in transcriptional regulation and evolving under medium to strong purifying selection. Liang *et al.* [10] used 17 grass genomes to predict the CNSs in the Poaceae lineage and found evidence of purifying selection acting on conserved sequences within the rice population. In addition, several studies have shown that individual CNSs can act as transcriptional enhancers that drive complex spatiotemporal expression patterns in animals [11, 12]. Moreover, some plant CNS studies have also shown that CNSs would be involved in *cis*-regulatory effects in many clades. Song *et al.* [13] developed a sensitive sequence alignment pipeline to identify maize CNSs in the Andropogoneae tribe and found that putative *cis*-regulatory sequences, transcription factor binding motifs (TFBMs), gene expression quantitative trait loci, and open chromatin regions were enriched in maize CNSs. Zhou *et al.* [14] systematically identified CNSs in 50 well-annotated Gramineae genomes using rice (*Oryza sativa*) as the reference and found rice CNSs significantly overlapped with putative active regulatory elements such as promoters, enhancers, and transcription factor binding sites. By searching nitrogen-fixing clade-specific CNSs among 25 nodulating species within the clade, Pereira *et al.* [15] identified several CNSs potentially involved in the regulation of genes that were required for nitrogen fixation. Hendelman *et al.* [16] predicted the Solanaceae CNSs and functionally validated one example of conserved *cis*-regulatory sequences control shared pleiotropy in distantly related Solanaceae species. However, the above-mentioned studies only identified CNSs using whole-genome alignments and did not further detect which CNSs were putative *cis*-regulatory elements.

Cucurbitaceae, which encompasses many important vegetables and medicinal plants, is one of the most genetically diverse plant families in the world. Many improved or new genome assemblies of Cucurbitaceae species have been produced over the past five years [13]. Among these, 12 closely related species, including *Cucumis sativus* [17], *Cucumis melo* [18], *Citrullus lanatus* [19], *Lagenaria siceraria* [20], *Cucurbita maxima* [21], *Cucurbita moschata* [21], *Cucurbita pepo* [22], *Cucurbita argyrosperma* [23]*, Benincasa hispida* [24]*, Luffa cylindrica* [25]*, Sechium edule* [26], and *Momordica charantia* [27], have been sequenced and assembled on pseudo-chromosomes.

However, there have been no reports on CNSs in Cucurbitaceae. To address this issue, we proposed a computational process for identifying CNSs from a group of closely related species and applied it to Cucurbitaceae. Based on these CNSs, we further detected Cucurbitaceae-wide putative *cis*-regulatory elements. To the best of our knowledge, this is the first pipeline for analysing clade-wide *cis*-regulatory elements. Finally, we released the CNSs and Cucurbitaceae-wide putative *cis*-regulatory elements through our public database, accessible via browser tools.

## Results

### Alignment of Cucurbitaceae to the cucumber reference genome

Due to cucumber’s small and compact genome in the cucurbitaceous family [28], we aligned 12 Cucurbitaceae genomes with the cucumber as the reference genome using LASTZ [29] and MULTIZ [30] pipelines. Although there were differences in genome size, sequencing quality, and assembly quality, we observed that the 12-way whole-genome alignment decreased with increasing divergence from cucumber, ranging from 88.64% (*C. melo*) to 55.70% (*L. cylindrica*), as illustrated in Table 1. The alignability ratios were much higher for the coding regions, ranging from 97.55% (*C. melo*) to 76.69% (*L. cylindrica*). The extremely high coverage of the coding regions substantiated the reliability of our alignment pipeline.

**Table 1:** Alignment coverage in 12-way comparison with cucumber as reference.

**Species**	**Nucleotides** ^**a**^	**Assembly**	**Reference**	**Total align (%)** ^**b**^	**CDS align (%)** ^**c**^	**Subs/Site** ^**d**^
*Cucumis sativus*	211 Mbp	V3	17			0
*Cucumis melo*	403 Mbp	V3.6.1	18	88.64	97.55	0.0979
*Lagenaria siceraria*	313 Mbp	V1	20	71.41	91.75	0.2662
*Citrullus lanatus*	354 Mbp	V2	19	70.8	91.84	0.2698
*Benincasa hispida*	859 Mbp	V1	24	69.42	85.97	0.3104
*Curcurbita maxima*	271 Mbp	V1.1	21	56.91	95.79	0.3632
*Cucurbita pepo*	263 Mbp	V4.1	22	57.24	95.95	0.3741
*Cucurbita moschata*	269 Mbp	V1	21	57.47	95.76	0.3753
*Momordica charantia*	293 Mbp	V1	27	61.56	95.94	0.3791
*Cucurbita argyrosperma*	229 Mbp	V2	23	56.77	95.47	0.3904
*Sechium edule*	608 Mbp	V1	26	58.31	95.48	0.3978
*Luffa cylindrica*	656 Mbp	V1	25	55.70	76.69	0.4572

a The genome size.

b The whole genome alignment coverage ratio with *C. sativus* as the reference.

c The alignment coverage ratio in protein coding sequences with *C. sativus* as the reference.

d The divergence from *C. sativus* based on the neutral tree of Fig. 1.

We obtained a phylogenetic tree based using the 12-way whole-genome alignment, as illustrated in Fig. 1. The topology of the tree was consistent with that observed in previous studies [20, 21, 23–26, 31], verifying the accuracy of our alignment. In addition, we also constructed 12 species phylogenetic trees based on 1148 single-copy protein-coding genes using the maximum likelihood (ML) and neighbor-joining (NJ) methods (Fig.S1, see online supplementary material). In the topology of the ML tree (Fig. S1a, see online supplementary material), *B. hispida* was found to be grouped with *C. sativus* and *C. melo,* which is different from the relationship based on the 12-way whole-genome alignment, but consistent with the relationship of previous studies [32, 33]. However, the NJ tree revealed that the *B. hispida* grouped with *L. siceraria* and *C. lanatus* (Fig. S1b, see online supplementary material), which is the same relationship observed in the phylogenetic tree based on the 12-way whole-genome alignment and the previous studies [20, 21, 23–26, 31, 34, 35]. The two topologies are possibly a result of differences in the sampling of genes set, the method used in the construction of the phylogenetic tree, and the inheritance of plastid and nucleus [32].

**Figure 1:**
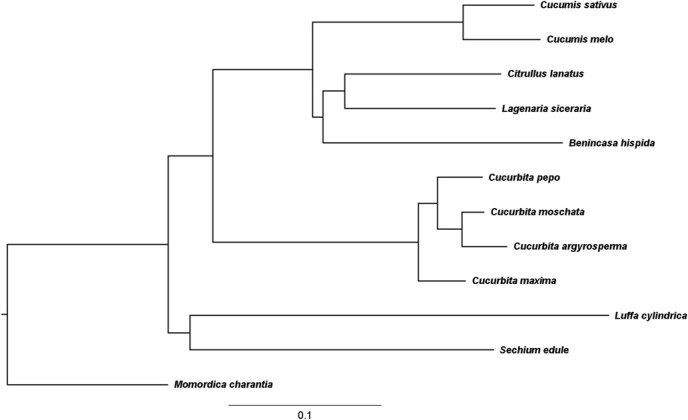
A phylogenetic tree of the relationships between species included in the 12-way whole-genome alignment. The neutral tree was based on four-fold degenerate sites sampled from cucumber chromosomes 1–7, with branches proportional to the listed scale and substitutions per site determined by PhyloFit [5]. The topology of the 12 species tree was constructed by MEGA [59] using 12-way whole-genome alignment with the Unweighted Pair Group Method with Arithmetic mean (UPGMA) method.

### Discrete, highly conserved sequences among the 12 Cucurbitaceae genomes

We used the phastCons [5] program and a two-state phyloHMM [36] (see Materials and Methods) to score conservation and identify the conserved regions in the 12-way alignment. Based on the 12-way alignment and phylogenetic tree (Fig. 1), we fitted the neutral (non-conserved) and conserved models (Fig. S2, see online supplementary material) using PhyloFit, a component of the PHAST package [5]. We used PhyloFit and phastCons to predict the conserved sequences. A smaller set of discrete elements representing the most highly conserved blocks of sequences was predicted and this was abbreviated as the most-Cons sequences. In total, we identified 143 580 most-Cons sequences in cucumber, with a mean length of 117 bp and median length of 74 bp (Table S1 and Fig. S3, see online supplementary material).

**Figure 2:**
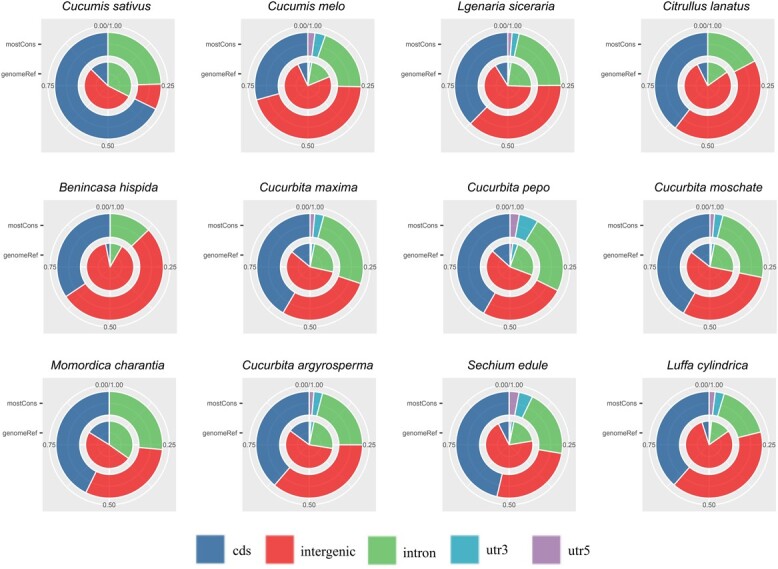
Composition of the most-Cons sequences predicted by phastCons in Cucurbitaceae genomes. The ‘genomeRef’ in the inner cycle and ‘mostCons’ in the outer cycle indicate the composition of the genome and most-Cons sequences predicted from phastCons, respectively. CDS: coding sequences; intergenic: intergenic regions; UTR: untranslated regions.

As shown in Fig. 2, we compared the predicted conserved sequences (outer cycle) to the normal composition of genomic features in each species (inner cycle). The composition of most-Cons sequences contrasted the normal distribution of the genome, revealing an expansion in the proportion of protein-coding and unannotated intergenic sequences. In general, the conserved sequences dataset is the starting point for further analysis and annotation of the conserved regions within Cucurbitaceae plants.

**Figure 3:**
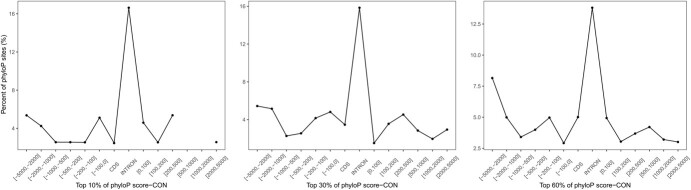
Percentage of sites predicted by phyloP for different sets of regions in the cucumber genome. CON: conservation site. Negative numbers indicate upstream of the gene, and the values in square brackets show the distance (base pairs) from the start codon. Positive numbers indicate downstream of the gene, and the values in square brackets show the distance (bases pairs) from the stop codon. CDS indicates the protein-coding sequences. All flanking regions that overlapped with nearby genes were removed.

We obtained the phyloP scores for alignment sites and counted the distribution trends around the genes upstream and downstream to observe the conservation patterns around the gene and its flanking sequences and distinguish the conserved elements (see Materials and Methods). The sequences of a gene and its flanking regions were divided into 14 classes. Fig. 3 illustrates the distribution of the top 10–60% fractions of phyloP scores in each class. Surprisingly, there were more conservation sites in the coding sequences than in other regions. In addition, we found that the region 100–500 bp and 2–5 kbp away from the start or stop codons harbored many highly conserved sites. A similar pattern was observed in the conserved non-coding sequences identified in cruciferous plants [9]. This implied that there were conserved sequences upstream and downstream of the genes, and that these likely colocalized with their nearby genes.

### Cucurbitaceae-wide putative *cis*-regulatory elements

We preserved 632 112 elements distributed in non-coding sequences with a minimal length of 20 bp and a minimal aligned identity of 80%, according to the annotations of the 12 genomes, from the predicted most-Cons sequences. These highly conserved non-coding sequences were designated as CNSs (Table S2, see online supplementary material). Sequence characteristics were explored to evaluate the accuracy of our predicted CNSs. We observed a sharp decline in A + T content near the border of the CNSs (Fig. S4a, see online supplementary material), a pattern similar to that observed in several other plants [7, 37]. We also observed nucleosome positioning around the center of the CNSs (see Materials and Methods) (Fig. S4b, see online supplementary material) that was similar to the pattern observed in four eudicot plant- [38] and monocot-specific CNSs [37]. We performed gene ontology (GO) functional enrichment analysis of the genes near the CNSs and identified that they were associated with GO terms related to organ or tissue development (Table S3, see online supplementary material). These results were consistent with those of previous studies [7, 37, 38] substantiating the accuracy of our CNS prediction.

In the previous section, we found that conserved sequences preserved co-localization with nearby genes in Cucurbitaceae genomes. Therefore, we wanted to determine whether CNSs maintained the conservation order with their nearby genes in Cucurbitaceae species. We then detected the collinear segments among 12 Cucurbitaceae species, as described in the Materials and Methods section. Similar to our previous study [39], we used conserved DNA segments across all 12 Cucurbitaceae genomes, rather than protein-coding genes, as genomic markers, and detected 43 133 collinear segments conserved across all 12 Cucurbitaceae plants (Table S4, see online supplementary material). We calculated the frequency distribution histogram of the distances between the putative *cis*-RCNEs and their closest genes in the collinear segments (Figs S5 and S6, see online supplementary material), suggesting that the 12 Cucurbitaceae genomes preserved the order of some CNSs and their adjacent genes. The conserved order of CNSs and their adjacent genes indicates the constraint of gene *cis*-regulation in Cucurbitaceae genomes. These CNSs, scattered in collinear segments, are presumed to be Cucurbitaceae-wide putative *cis*-regulatory elements. Guided by 12-way collinear segments, we identified 16 552 Cucurbitaceae-wide putative *cis*-regulatory elements in all 12 species (Table S5, see online supplementary material) and named these as *cis-*RCNEs. These putative *cis*-RCNEs were located in the 12-way collinear segments identified using i-AdHore [40] with multiple alignment anchors as markers, indicating that the putative *cis*-RCNEs were highly conserved and that they co-localized with their potential nearby target genes across all 12 Cucurbitaceae species.

### Sequence properties and function of putative *cis-*RCNEs

**Figure 4:**
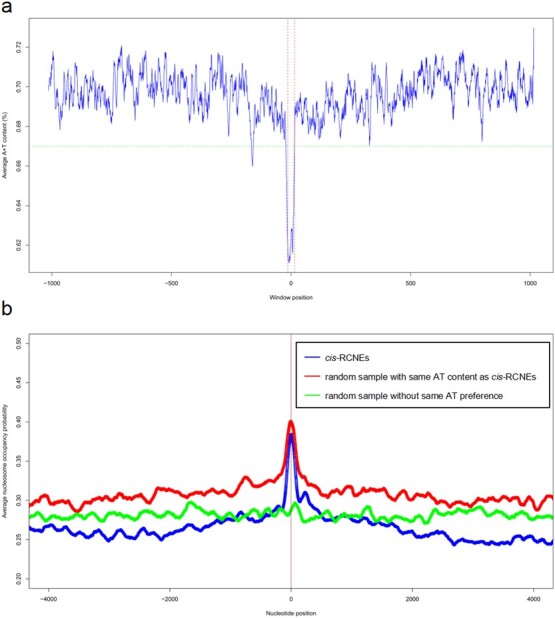
Distribution of A + T content and nucleosome occupancy probabilities in the flanking regions and within putative *cis*-RCNEs with the cucumber genome as reference. **a** The distribution of A + T content. Green line: Average A + T content in the cucumber genome. Red lines: A + T content inside *cis*-RCNEs (30 bp from the center of each *cis*-RCNE was considered as mentioned in the methodology) acquired through moving window analysis. **b** The distribution of nucleosome occupancy probabilities. The brown line represents the center of each *cis*-RCNE and the center of the random samples. The blue curve shows the nucleosome occupancy probabilities of *cis-*RCNEs, the red curve is a random sample with the same A + T content as *cis*-RCNEs, and the green curve is a random sample without a specific A + T content preference.

The A + T content inside the putative *cis-*RCNEs and their flanking regions was determined, as described in the Materials and Methods section, to explore the sequence characteristics of the putative *cis-*RCNEs. A sharp decline in the A + T content was observed around the center of the putative *cis*-RCNEs (Fig. 4a). A significant difference in the A + T content was observed between the center of the putative *cis*-RCNEs and their flanking regions (*t*-test, *P*-value = 6.87e-17). A similar pattern was also observed in four eudicot plant- [38] and monocot-specific CNSs [37].

Generally, the G + C content affects DNA topology and nucleosome positioning. The nucleosome positioning pattern facilitates the access of transcription factors to their target sites [41]. Jansen and Verstrepen [42] found that A + T-rich sequences in *Saccharomyces cerevisiae* had a low tendency to form nucleosomes. Therefore, the A + T drop near the center of CNSs may contribute to nucleosome formation. Nucleosome prediction software [43] was used to calculate the probability of nucleosome occupancy by putative *cis*-RCNEs and their flanking regions. Compared to the two control samples, a significant peak in the center of putative *cis*-RCNEs potentiated nucleosome positioning (Fig. 4b). A previous review proposed that nucleosome occupancy is closely related to the regulation of gene expression [44].

The nucleosome positioning pattern has also been observed in humans [44], four eudicot plant CNSs [38], and monocot-specific CNSs [37]. Nucleosome high-occupancy was calculated based on CNS sequences, which are positively correlated with the G + C content, indicating high G + C content. Therefore, our findings are consistent with those of Lu *et al.* [45]. Tillo *et al.* [44] proposed that nucleosome occupancy of regulatory sequences might keep them masked with nucleosomes, unless they are used. This was reported as nucleosome depletion by Bai and Morozov [41]. This nucleosome positioning pattern minimizes instances of inappropriate utilization and aberrant transcription from open chromatin [44] and reflects dynamic regulation [41]. Based on these observations, we presumed that *cis*-RCNEs are involved in regulating the expression of their putative target genes.

According to previous related studies [4–6, 46–48], the genes closest to CNSs are considered their candidate target genes. Therefore, we assumed that the adjacent genes flanked the 5 kbp range of each *cis-*RCNE. If adjacent genes were located in 12-way collinear segments, we predicted them as candidate targets. Functional enrichment analysis of these *cis*-RCNE putative target genes identified GO terms including the regulation of biological processes and plant organ development (Table S6, see online supplementary material).

### Low divergence in Cucurbitaceae-wide putative *cis*-RCNEs

The highly conserved nature of *cis*-RCNEs suggests that mutations within them could have deleterious effects. In this case a lower polymorphism rate in the putative *cis-*RCNE regions at the population level can be expected. We assessed sequence polymorphism patterns within the cucumber population. We downloaded all single nucleotide polymorphism (SNP) datasets of 115 cucumber lines [49] (the cucumber V2 genome version). The V2 cucumber genome was aligned to the V3 genome using LASTZ [29] with the same parameters to map the sequence polymorphism within the 115 cucumber lines from the V2 to V3 cucumber genome. Indeed, the frequency of SNPs in the putative *cis*-RCNEs (7.58 SNPs per kb) was significantly lower than that in the whole-genome average (16.12 SNPs per kb) (Chi-square test, *P*-value = 1.25e-95), and it was comparable to the average level in coding regions (7.78 SNPs per kb) (Table 2). These results suggest that putative *cis*-RCNEs have been under strong selection constraints, during long-term evolution and at recent intraspecific levels.

**Table 2:** Statistics of SNPs located in putative *cis-*RCNEs and other genomic regions with the cucumber V3 genome as the reference.

	**SNP** ^**a**^	**Total length (bp)**	**SNP per kb**	**Chi-square** ^*^ **(vs whole genome)**	**Chi-square** ^*^ **(vs coding region)**
***cis*-RCNEs**	712	93 876	7.58	*P*-value =1.25e-95	*P*-value = 0.5052
**CNSs**	24 413	3 094 556	7.89	*P*-value = 0	*P*-value = 0.0415
**Most-Cons elements**	118 398	16 910 462	7.00	*P*-value = 0	*P*-value = 5.23e-187
**Coding region**	209 563	26 931 795	7.78	*P*-value = 0	
**Protein-coding genes**	1 170 268	96 992 618	12.07	*P*-value = 0	*P*-value = 0
**Whole genome**	3 400 665	210 974 704	16.12		*P*-value = 0

^*^ *P*-value was calculated by *chi-*square test

a Sequence polymorphism dataset within 115 cucumber lines from [49]

### Putative *cis*-RCNEs and their potential target genes

**Figure 5:**
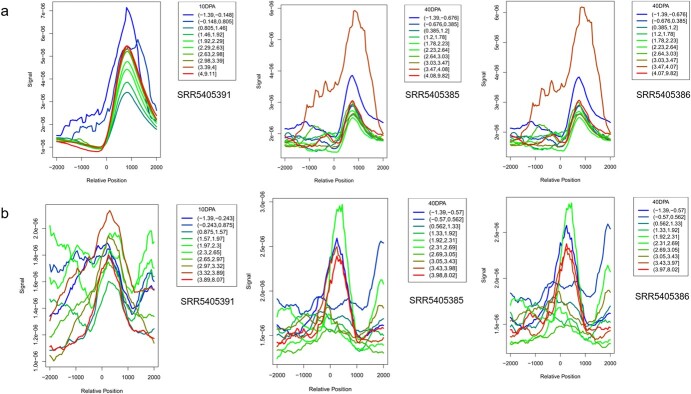
The H3K27me3 signal near the center of promoters and putative *cis*-RCNEs during *Cucumis sativus* fruit development stages. (**a**) Promoters, (**b**) putative *cis*-RCNEs. The line plot indicates the H3K27me3 signal according to expression strength. Line colors reflect the expression level as log(RPKM) (reads per kilobase of transcript, per million mapped reads), whereas line shapes correspond to the averaged H3K27me3 signals across the putative *cis*-RCNEs. Along the x-axis is the location of the signal relative to the *cis*-RCNEs. Zero in the x-lab indicates the center of promoters or *cis*-RCNEs. Fresh fruits 10 days post-anthesis (10DPA) and fresh fruits 40 days post-anthesis (40DPA) were considered for the experiments. All counts with the coding regions were removed from the BAM files to avoid the effect of the coding sequences on the *cis-*RCNEs.

Based on the proximity principle, we predicted 3 271 potential regulatory pairs in the cucumber genome as a reference (Table S7, see online supplementary material). We explored the roles of these regulatory pairs through integrative analysis using public datasets. All raw cucumber RNA sequencing (RNA-seq) and chromatin immunoprecipitation (ChIP) sequencing (ChIP-seq) reads from fruitENCODE [50] were downloaded. Integrative analysis between RNA-seq and ChIP-seq was performed with samples collected from several cucumber fruit development stages, as described in the Materials and Methods section. As illustrated in Fig. 5b, a strong H3K27me3 signal peak was observed near the center of putative *cis*-RCNEs in different fruit development stages, including the 10 and 40 day post-anthesis stage. Interestingly, we also found a high H3K27me3 signal near the promoter regions (Fig. 5a) which is similar to the *A. thaliana* study [51]. We speculated that these putative *cis*-RCNEs could regulate gene expression in a manner similar to that of promoters, and some of the *cis*-RCNEs were also located in the upstream regions of genes. In contrast, no such pattern was observed in the shuffling putative *cis*-RCNE tests (Fig. S7, see online supplementary material).

Although recent studies have reported that maize CNSs [13] and rice CNSs [14] were enriched in potential active regulatory sequence such as H3K9ac and open chromatin, rice CNSs were further classified into 20 types according to conservation states in which conservation states 7–15 were associated with repressive chromatin states marked by K3K27me3 [14]. Interestingly, we also observed that the putative *cis*-RCNEs, a subset of the CNSs in Cucurbitaceae, were enriched in H3K27me3. Our putative *cis*-RCNEs were detected from the CNSs covered by the 12-way collinear segments using multiple alignment anchors as markers [39], meaning that the putative *cis*-RCNEs and the targets genes were maintained in the 12-way syntenic blocks. Syntenic blocks like this have been reported in metazoan genomes named genomic regulatory blocks [1], where genes proposed as targets of CNSs regulation were marked by broad polycomb repression in an inactive state [52]. Therefore, we speculate that our putative *cis*-RCNEs belong to the condition.

Integrating the RNA-seq and ChIP-seq data, two signals were observed to verify the regulatory relationship between putative *cis*-RCNEs and their nearby target genes. If one *cis*-RCNE overlapped with the H3K27me3 binding peak at the regulatory signal between any two fruit development stages, we checked the expression of nearby target genes under the two conditions. If a gene showed significant differential expression, we predicted that a *cis*-RCNE could regulate this gene between the two fruit development stages. Thus, we predicted 98 *cis*-RCNE-target regulatory pairs across three cucumber fruit development stages (Table S8, see online supplementary material). For the genes potentially regulated by these putative *cis*-RCNEs, functional enrichment analysis showed that cell wall metabolism, cell wall biogenesis, and DNA/RNA binding were involved in the fruit development stages (Table S9, see online supplementary material).

For example, CsaV3_5G027790 encodes a xyloglucan endotransglucosylase/hydrolase associated with cell wall biogenesis (GO:0042546) located on chromosome 5 of the cucumber genome. We identified one putative *cis*-RCNE named *cis*-RCNEs_ID_chr5.8522 (length, 40 bp), 6097 bp downstream of CsaV3_5G027790. One H3K27me3 binding peak across *cis*-RCNEs _ID_chr5.8522 was detected at 40 days post-anthesis but not in the leaves. Compared with the expression in the leaves, the expression of CsaV3_5G027790 at 40 days post-anthesis significantly increased with logFC (fold change) of 1.18 and false discovery rate (FDR) of 3.10e-05. However, further experimental evidence is required to validate the regulatory roles of these putative *cis*-RCNEs.

## Discussion

Our pipeline integrated several tools, including LASTZ [29] and MULTIZ [30] (whole-genome alignment), phastCons [5] (conservation scoring), and i-AdHore [40] (collinear segments detection), to identify *cis-*regulatory elements. Using our localized pipeline, we identified 632 112 CNSs among 12 Cucurbitaceae genomes based on a 12-way whole-genome alignment. We identified 16 552 Cucurbitaceae-wide putative *cis*-RCNEs using collinearity information. We further predicted 3 271 potential regulatory pairs in the cucumber genome. Ninety-eight *cis*-RCNE-target regulatory pairs were validated using RNA-seq and ChIP-seq datasets across three cucumber fruit development stages. In addition, we constructed a database and a server to access the datum (http://cmb.bnu.edu.cn/cisRCNEs_cucurbit/).

To verify the scalability of the pipeline of *cis*-RCNEs identification, we performed the same analysis in six Solanaceae plants (data source in Table S10 and the phylogenetic tree in Fig. S8, see online supplementary material). Consistent with previous results of Cucurbitaceae CNSs, we observed similar patterns including high coverage of coding sequences (Table S11, see online supplementary material), the composition of most-Cons sequences contrasted with the normal distribution of the ref genome (Fig. S9, see online supplementary material), the large proportion of conserved noncoding sites scattered in intron and flanking upstream or downstream (Fig. S10, see online supplementary material), AT-drop and highly nucleosome occupancy near the center of Solanaceae *cis*-RCNEs, and a significant H3K27me3 signal in the center of putative Solanaceae *cis*-RCNEs (Fig. S11, see online supplementary material). Moreover, we observed that the H3K27me3 signal in the center of putative Solanaceae *cis*-RCNEs could be removed by a histone lysine demethylase that specifically demethylates H3K27 methylation (Fig. S12, see online supplementary material). The results of the six Solanaceae plants are accessible at http://cmb.bnu.edu.cn/cisRCNEs_solanaceae/.

According to our survey, highly conserved sequences account for 8.01% of the cucumber genome. The coverage of these conserved sequences in the cucumber genome was 3–8% larger than that in animal genomes [5]. This may be due to the small divergence distance of the 12 included Cucurbitaceae species, i.e. nearly 31 and 26 million years [22]. Interestingly, we found that the composition of CNEs deviated from the normal composition of cucumber genome, indicating substantial enrichment in the proportion of protein-coding sequences. Even though nearly three-quarters of all conserved sequences were located in protein-coding regions, a considerable number of elements were still present in intergenic regions. There is no known DNA-level annotation for these elements, suggesting that a substantial amount of undiscovered functional DNA elements are present in cucumber and other Cucurbitaceae genomes. Previous comparative genomic studies also observed this pattern [8, 9].

Identification of collinear segments generally requires gene order conservation and orientation [40, 53]. Unfortunately, many genomic events, including whole-genome duplication, short DNA segment reshuffling by mobile elements, and horizontal gene transfer, destroy gene order, especially in plant genomes. Therefore, collinearity detection using genes as genome-wide markers for whole-genome comparisons in plants is challenging. An increasing number of comparative genome studies have demonstrated that smaller units, such as evolutionarily stable domains or segments, are better genome-wide markers in whole-genome comparisons [54]. We demonstrated the feasibility of utilizing these DNA segments conserved across several related genomes as genomic markers for detecting collinear segments.

Our pipeline used one conserved DNA segment to detect collinearity and identify clade-wide *cis*-regulatory elements in several closely related species. These collinear segments contained genomic information for coding and non-coding sequences. Additionally, it contained a gene body and adjacent flanking regulatory regions. Our observations in the Cucurbitaceae family verified that genes and their surrounding regulatory elements may have selectively retained a consistent order during long-term evolution.

However, our study had several limitations. First, the multiple alignment anchors used to detect the collinear segments needed to be carefully screened to integrate phylogenetic and evolutionary model analyses, such as BinCons [55] or GERP [56], sequence character, and lineage-specific information. Second, the phastCons [5] models for identifying conserved DNA sequences were merely fitted with the coding sequences, which may have biased the results. Phylogenetic models should be constructed using non-coding sequences to combat this. In addition, further experimental evidences are necessary to validate the identified *cis-*RCNEs.

In summary, we proposed a computational pipeline to identify clade-wide putative *cis-*regulatory elements and characterize their potential function within plant genomes based on a 12-way whole-genome alignment. Our results may benefit the biological functional annotation of non-coding sequences in Cucurbitaceae plants.

## Materials and methods

### Data source

The present study used 12 Cucurbitaceae genomes, including *C. sativus* [17], *C. melo* [18], *C. lanatus* [19], *L. siceraria* [20], *C. maxima* [21], *C. moschata* [21], *C. pepo* [22], *C. argyrosperma* [23]*, B. hispida* [24]*, L. cylindrica* [25]*, S. edule* [26], and *M. charantia* [27], assembled on pseudo-chromosomes. We downloaded RNA-seq and ChIP-seq raw data from NCBI (SRP102870) to validate the gene regulatory modules. The structure and functional annotations of the cucumber genome were obtained from http://cucurbitgenomics.org/. Table 3 summarizes the genomes, structure, functional annotations, and public sequence read archive datasets for validating gene regulatory modules.

**Table 3:** Genome assembly versions, annotation resources, RNA-seq, and ChIP-seq data.

**Species**	**Data type**	**Assembly**	**Annotation**	**Release Date**	**URL**
***Cucumis sativus***	Genome	V3	V3	18 June 2019	http://cucurbitgenomics.org/ftp/genome/cucumber/Chinese_long/v3/
***Cucumis melo***	Genome	V3.6.1	V4.0	24 May 2018	ftp://cucurbitgenomics.org/pub/cucurbit/genome/melon/v3.6.1/
***Citrullus lanatus***	Genome	V2	V2	11 January 2019	http://cucurbitgenomics.org/ftp/genome/watermelon/97103/v2/
***Lagenaria siceraria***	Genome	V1	V1	23 September 2017	ftp://cucurbitgenomics.org/pub/cucurbit/genome/Lagenaria_siceraria/
***Benincasa hispida***	Genome	V1	V1	14 November 2019	http://cucurbitgenomics.org/ftp/genome/wax_gourd/
***Curcurbita maxima***	Genome	V1.1	V1.1	14 September 2017	ftp://cucurbitgenomics.org/pub/cucurbit/genome/Cucurbita_maxima/
***Cucurbita moschata***	Genome	V1	V1	14 September 2017	ftp://cucurbitgenomics.org/pub/cucurbit/genome/Cucurbita_moschata/
***Cucurbita pepo***	Genome	V4.1	V4.1	7 November 2017	ftp://cucurbitgenomics.org/pub/cucurbit/genome/Cucurbita_pepo/
***Momordica charantia***	Genome	V1	V1	23 June 2020	https://ddbj.nig.ac.jp/public/ddbj_database/wgs/BL/BLBB.gz
***Cucurbita argyrosperma***	Genome	V2	V2	1 April 2019	http://cucurbitgenomics.org/ftp/genome/Cucurbita_argyrosperma/
***Sechium edule***	Genome	V1	V1	31 January 2021	http://cucurbitgenomics.org/ftp/genome/chayote/
***Luffa cylindrica***	Genome	V1	V1	1 August 2020	https://ftp.ncbi.nlm.nih.gov/genomes/genbank/plant/Luffa_aegyptiaca/latest_assembly_versions/GCA_017139565.1_ASM1713956v1/
***Cucumis sativus***	RNAseq/ChIP-seq	/	/	1 April 2017	https://trace.ncbi.nlm.nih.gov/Traces/study/?acc=SRP102870

### 12-way whole-genome alignment

A pairwise alignment was used to generate a 12-way whole-genome alignment against the cucumber reference genome. First, RepeatMasker [57] (species = ‘Cucurbitaceae’) was used to remove repetitive regions before pairwise alignment. Then, LASTZ [29] was used to perform pairwise alignment with the following parameters: ‘gapped, inner=2000, xdrop=9400, gapped thresh=3000, hsp thresh=2200’. The LASTZ output relating the query to the reference was then linked into longer chains of contiguous alignment using axtChain [58]. The alignment chains were sorted using chainNet [58], which filtered only the single best-aligned chain and maximized the coverage across the reference genome. Then the nets were converted into multiple alignment files. MULTIZ [30] merged all pairwise alignments into a 12-way whole-genome alignment against the cucumber reference genome (R = 30, M = 20). The 12-way multiple alignment could be viewed as a series of conserved blocks across all 12 species and contained the best match within the cucumber reference. We termed these 12-way blocks conserved across the 12 Cucurbitaceae genomes as the ‘multiple alignment anchors’.

We reconstructed three phylogenic trees of 12 Cucurbitaceae plants: (i) using the UPGMA method in MAGAX [59] and a supermatrix concatenated by 143 580 mostCons elements conserved through all 12 Cucurbitaceae plants from 12-way whole-genome alignments; (ii) using the maximum likelihood method by RAxML-NG v0.9.0 [60] and a supermatrix concatenated by 1148 single-copy protein sequences identified by OrthoFinder v2.5.4 [61] for the comparison of protein-coding sequences in 12 plants, with 1000 bootstrap replicates under the General Time Reversiable model; and (iii) using the neighbor joining method in MEGAX and the supermatrix concatenated by 1148 single-copy protein sequences identified by OrthoFinder, with the 1000 bootstrap replicates under the Maximum composite Likelihood model.

### Scoring conservation with 12-way alignment

We used phyloFit [5] to fit the two phylogenetic models to compute the conservation among the 12-way alignment. With the cucumber genome as the reference, one model was fitted from all four-fold degenerate sites; these are also known as the neutral or non-conserved models, and the other model was fitted from all first and second codon sites, also known as the conserved model.

We used phyloP [62] to compute site-by-site scores of measured evolutionary conservation based on the 12-way alignment and a model of neutral evolution obtained from four-fold-degenerate sites with the cucumber genome as the reference. Here, we utilized PhyloP with the default smoothed-particle hydrodynamics (SPH) method to compute conservation *P-*values based on alignment and a model of neutral evolution using independent hypothesis tests (−log *P-*values) at individual nucleotides. We excluded all upstream and downstream regions that overlapped with the gene body to avoid overlap between the regions and their nearby genes.

### *Cis*-RCNE identification

Identifying putative *cis-*RCNEs consisted of detecting CNSs and predicting clade-wide putative *cis*-regulatory elements. The detection of CNSs was similar to a previous study [39]. First, neutral and conserved models were used with phastCons [5] to predict highly conserved sequences from the 12-way alignment. The parameters required by phastCons were the same as those in previous analyses focusing on *A. thaliana* [8], with an expected coverage of 0.2 and expected length of 80 bp. Then, the highly conserved sequences in the coding regions were filtered out, and only the CNSs remained. Therefore, CNSs were identified from pairwise collinear segments. Predicting putative *cis*-regulatory elements is an innovative process. First, we applied i-ADHoRe [40], which uses multiple alignment anchors as markers [39], to identify collinear segments among the 12 Cucurbitaceae species. Putative *cis*-RCNEs were detected from the CNSs covered by the 12-way collinear segments and were likely colocalized with potential nearby target genes.

### Calculating A + T content and nucleosome occupancy probability in putative *cis*-RCNEs and their flanking regions

One analysis to characterize Cucurbitaceae-wide putative *cis*-regulatory elements was carried out by exploring the A + T content in 1000 base flanking regions and the center (30 bases) of *cis-*RCNEs by moving window analysis using a 10-base window with one-base step size. *Cis-*RCNEs with flanking regions that ran into coding regions were removed from the analysis. Statistical significance was assessed using *t-*test.

Another analysis to characterize Cucurbitaceae-wide putative *cis*-regulatory elements evaluated the nucleosome occupancy probabilities for putative *cis*-RCNEs and their flanking regions. We adopted Kaplan’s probabilistic model [43] of sequence preferences of nucleosome regions by considering a 5000 bases region on each side starting from the center of the putative *cis*-RCNEs. The average nucleosome occupancy probability was then computed for both sides of each nucleotide site along the length of the sequences (in total, 10 001 sites). The same analysis was carried out for a random sample with the same length and A + T content as the putative *cis*-RCNEs, and for a random sample with no specific A + T preference. These random samples contained the same number of sequences as the putative *cis*-RCNEs, with additional extending flanking regions. All sequences were extracted from non-coding regions of the cucumber genome. Statistical significance was determined using *t-*test.

### Functional enrichment analysis for the potential target genes of putative *cis-*RCNEs

Genes close to a *cis*-RCNE located in 12-way collinear segments were considered potential target genes. Functional enrichment analysis was performed using an Ontologizer [63]. The running parameters were set as follows: -g go.obo.txt (version 1.2; data version: release on 27 October 2016); −c: Parent–Child-Union. Functional annotations of protein-coding genes in the cucumber genome were obtained from our previous work [39].

### Processing and analysing ChIP-seq and RNA-seq datasets

FruitENCODE [50] constructed multidimensional datasets for the functional genomics of fleshy fruit. We analysed the ChIP-seq and RNA-seq data of cucumber from fruitENCODE to profile their tissue-specific/time-specific DNA methylation and transcriptome profiles. RNA-seq reads were mapped to the reference genome using Hisat2 [64]. ChIP-seq reads were uniquely mapped to the reference genomes using bowtie2 [65] version 2.2.9, with default settings. Peak calling of ChIP-seq data was performed using the systemPipeR [66]. Integrative analysis between RNA-seq and ChIP-seq data and differential expression analysis were performed using edgeR [67].

## Acknowledgments

This work was supported by the National Natural Science Foundation of China (grant number: 31571361).

## Author contributions

E.P. contributed the central idea. H.S. and E.P. designed the study and wrote the manuscript. H.S. analysed most of the data. Z.Z. and K.L. refined the central idea. Q.W. analysed the RNA-seq data. All authors discussed the results and revised the manuscript.

## Data availability

All identified putative *cis*-RCNEs, main computational pipelines, and other annotation results were released and are accessible at http://cmb.bnu.edu.cn/cisRCNEs_cucurbit/.

## Conflict of interest

The authors declare that they have no conflicts of interest.

## Supplementary data

Supplementary data is available at *Horticulture Research* online.

## Supplementary Material

Web_Material_uhad038
