# Supplementary material for: Identification of clade-wide putative cis-regulatory elements from conserved non-coding sequences in Cucurbitaceae genomes
Source: Hortic Res. 2023 Feb 28;10(4):uhad038. doi: 10.1093/hr/uhad038 (PMC10548412; doi:10.1093/hr/uhad038)
Supplement: Web_Material_uhad038 [file web_material_uhad038.zip › Supplementary-20221227.docx]

Identification of Clade-wide Putative *Cis*-regulatory Elements from Conserved Non-coding Sequences in Cucurbitaceous Genomes

Hongtao Song^1^, Qi Wang^1^, Kui Lin^1^, Zhonghua Zhang^2^ and Erli Pang^1*^

^1^ MOE Key Laboratory for Biodiversity Science and Ecological Engineering and Beijing Key Laboratory of Gene Resource and Molecular Development, College of Life Sciences, Beijing Normal University, Beijing 100875, China

^2^ College of Horticulture, Qingdao Agricultural University, Qingdao 266109, China

*** Correspondence:**

pangerli@bnu.edu.cn

Keywords: Cucurbitaceae, conserved non-coding sequences, clade-wide *cis*-regulator, whole genome alignment, collinearity

## Supplementary figures and tables

Fig. S1 The phylogenetic trees of 12 cucurbitaceous species constructed by protein-coding genes

Fig. S2 Average phylogenetic trees describing branch length evaluated by all conserved sites and non-conserved sites in Cucurbitaceous plants

Fig. S3 Length summary of various elements in cucumber

Fig. S4 Distribution of A+T content and nucleosome occupancy probabilities in the flanking regions and within CNSs with cucumber genome as a reference

Fig. S5 Frequency distribution histogram of distances between putative *cis-*RCNE and its closest genes

Fig. S6 One example screenshot of *cis-*RCNEs, collinear segments and nearby genes models from Jbrowse database

Fig. S7 Integrative analysis using 1000 times of shuffling DNA elements in cucumber

Fig. S8 A phylogenetic tree of the relationships between species included in the 6-way whole-genome alignment

Fig. S9 Composition of the most-Cons sequences predicted by phastCons in S olanaceae genomes

Fig. S10 Percentage of sites predicted by phyloP for different sets of regions in the tomato genome

Fig. S11 Distribution of A+T content and nucleosome occupancy probabilities in the flanking regions and within putative *cis*-RCNEs with the tomato genome as reference

Fig. S12 The H3K27me3 signal near the center of putative *cis*-RCNEs in regulating fruit ripening-regulated genes of tomato

-------------------------------------------------------------------------------------------------------

Table S1: All mostCons-elements in cucumber

Table S2: All mostCons-elements in noncoding region of cucumber

Table S3: Functional enrichment of putative CNSs targets genes across three cucumber fruit development stages

Table S4: 12-way collinear segments in all 12 Cucurbitaceous genomes

Table S5: All putative *cis*-RCNEs in 12 Cucurbitaceous genomes

Table S6: Functional enrichment of the adjacent genes of putative *cis*-RCNEs in cucumber

Table S7: 3,271 potential *cis*-RCNE-gene regulatory pairs in cucumber

Table S8: 98 *cis*-RCNE-target pairs across three cucumber fruit development stages

Table S9: Functional enrichment of putative *cis*-RCNEs targets genes across three cucumber fruit development stages

Table S10: Genome assembly versions, annotation resources, RNA-seq, and ChIP-seq data used in *cis*-RCNEs identification in Solanaceae plants

Table S11: Alignment coverage in 6-way comparison with tomato as reference

--------------------------------------------------------------------------------------------------------

Website: http://cmb.bnu.edu.cn/cisRCNEs_cucurbit/index.html

All the putative *cis*-RCNEs elements identification in Cucurbitaceous species

elements display based on JBrowse.

Website: http://cmb.bnu.edu.cn/cisRCNEs_solanaceae/index.html

All the putative *cis*-RCNEs elements identification in Solanaceae species

elements display based on JBrowse.

**Fig. S1 The phylogenetic trees of 12 Cucurbitaceous species constructed by protein-coding genes**

(a) ML tree constructed with the maximum likelihood method by RAxML-NG^1^ and a supermatrix concatenated by 1,148 single-copy protein sequences identified by OrthoFinder^2^ v2.5.4 with the number of bootstrap replicates set to 1000 under the General Time Reversiable model; (b) NJ tree constructed with the neighbor joining method by MEGAX^3^ software and the supermatrix same to (a), with the number of bootstrap replicates set to 1000 under the Maximum composite Likelihood model

**
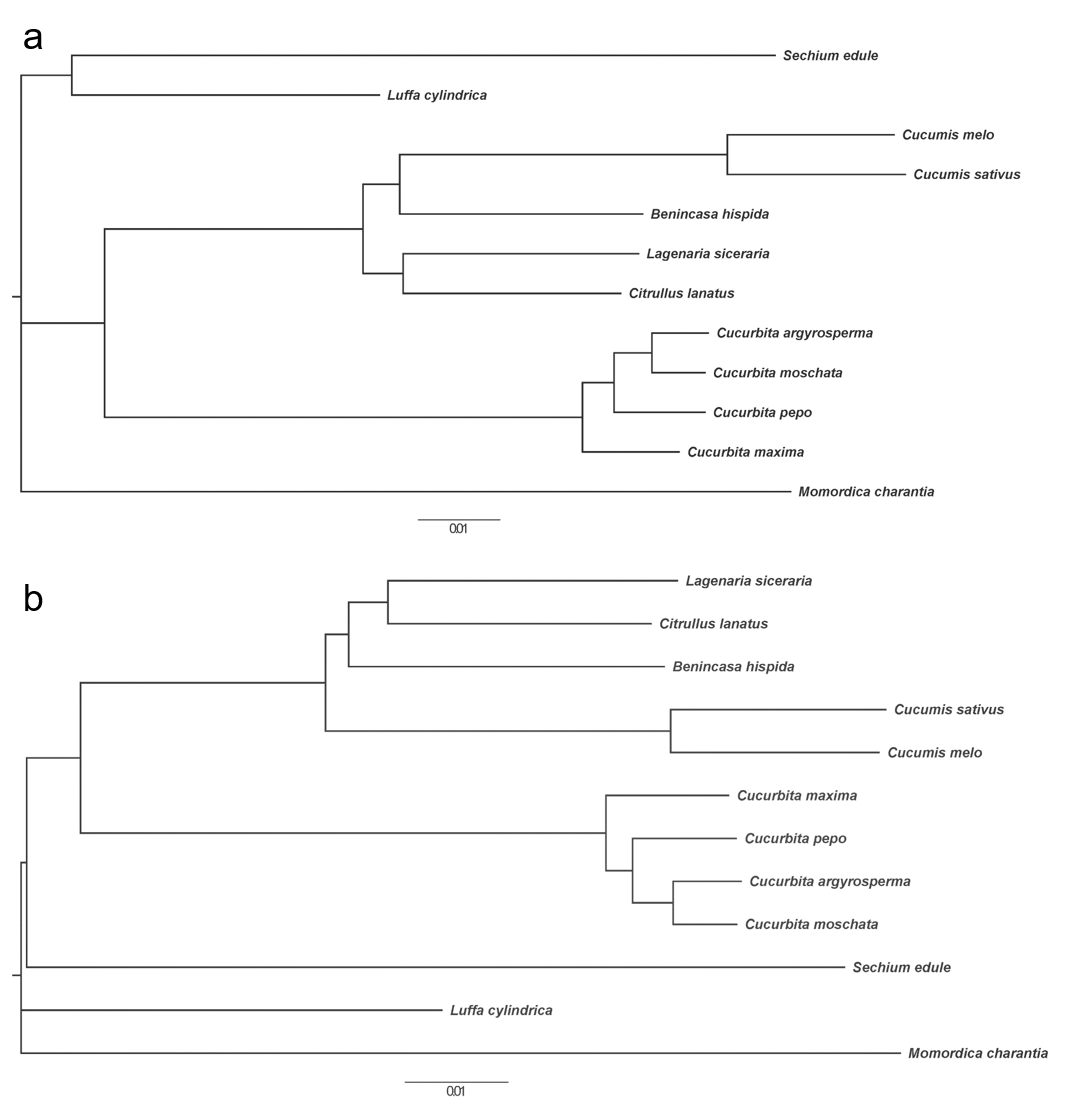
**

**Fig. S2 Average phylogenetic trees describing branch length evaluated by all conserved sites (a) and non-conserved sites (b) in Cucurbitaceous plants**

The conserved tree is based on 1^st^ and 2^nd^ codon sites, and non-conserved tree is based on 4-fold degenerate sites sampled from cucumber chr1~7 with branches proportional to the listed scale, with substitutions per site determined by PhyloFit^4^. The topology of the 12 species tree was constructed by MEGA^3^ using the 12-way whole genome alignments data-set with UPGMA method.


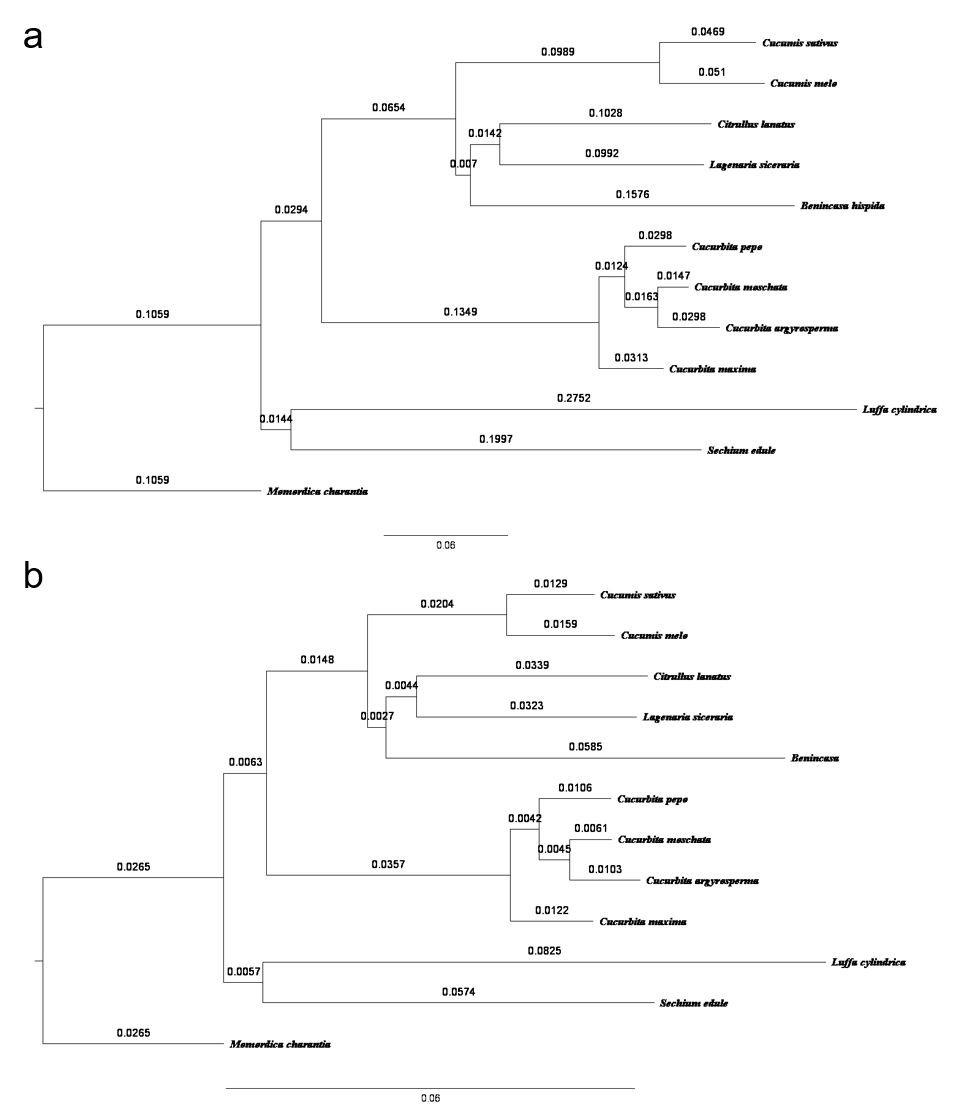


**Fig. S3 Length summary of various elements in cucumber**

Genes indicated the protein-coding genes in cucumber genome; MAAs indicated the multiple alignment anchors; mostCons indicated the mostCons elements identified by phastCons; CNSs indicated the mostCons elements in noncoding regions; Coll indicated the 12-way collinear segments among 12 Cucurbit species; *cis-*RCNEs indicated *cis*-regulatory conserved non-coding elements candidates predicted by our pipeline.


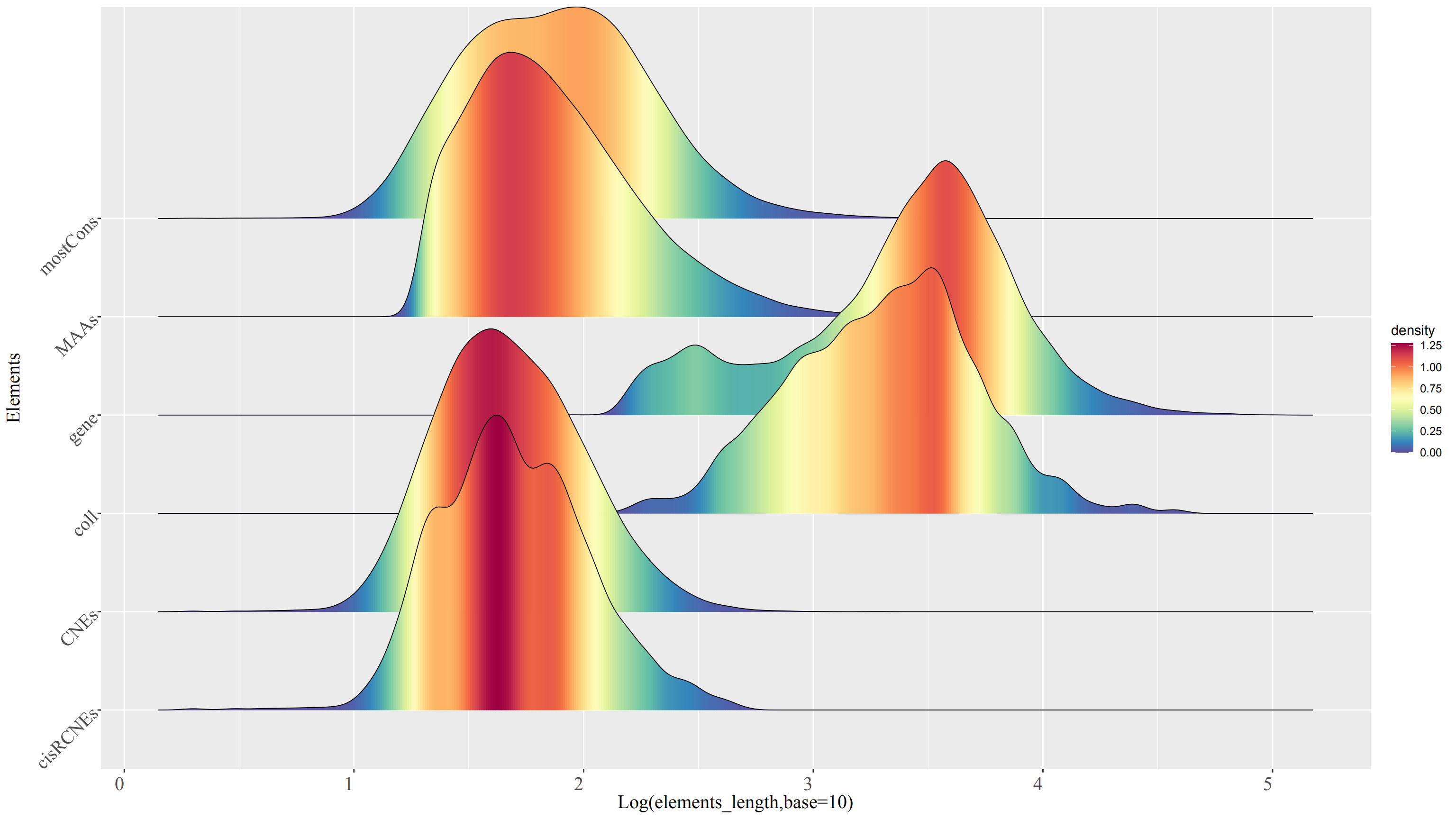


**Fig. S4. Distribution of A+T content and nucleosome occupancy probabilities in the flanking regions and within putative CNSs with cucumber genome as a reference**

1. Distribution of A+T content. Green line: average A+T content in cucumber genome; Red lines: A+T content inside CNSs (30 bp from the center of each *cis*-RCNEs was considered as mentioned in the methodology) acquired through moving window analysis; (b) Distribution of nucleosome occupancy probabilities. The brown line represents the center of each CNSs and also the center of the random samples. The blue curve shows the nucleosome occupancy probabilities of the CNSs, the red curve is a random sample with the same AT content as CNSs, and the green curve is the random sample without specific AT content preference.

**
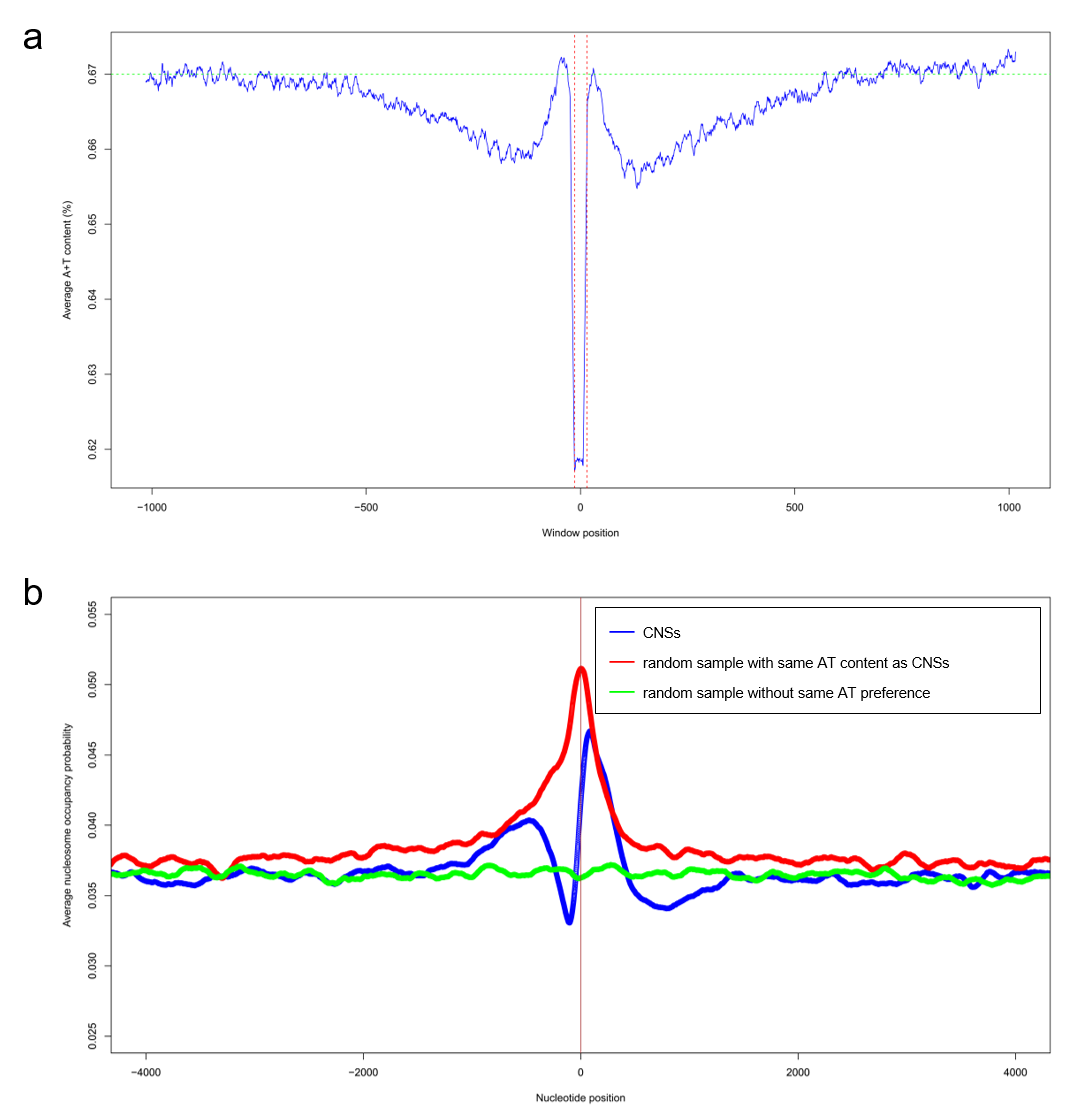
**

**Fig. S5 Frequency distribution histogram of distances between putative *cis-*RCNE and its closest genes in cucumber**


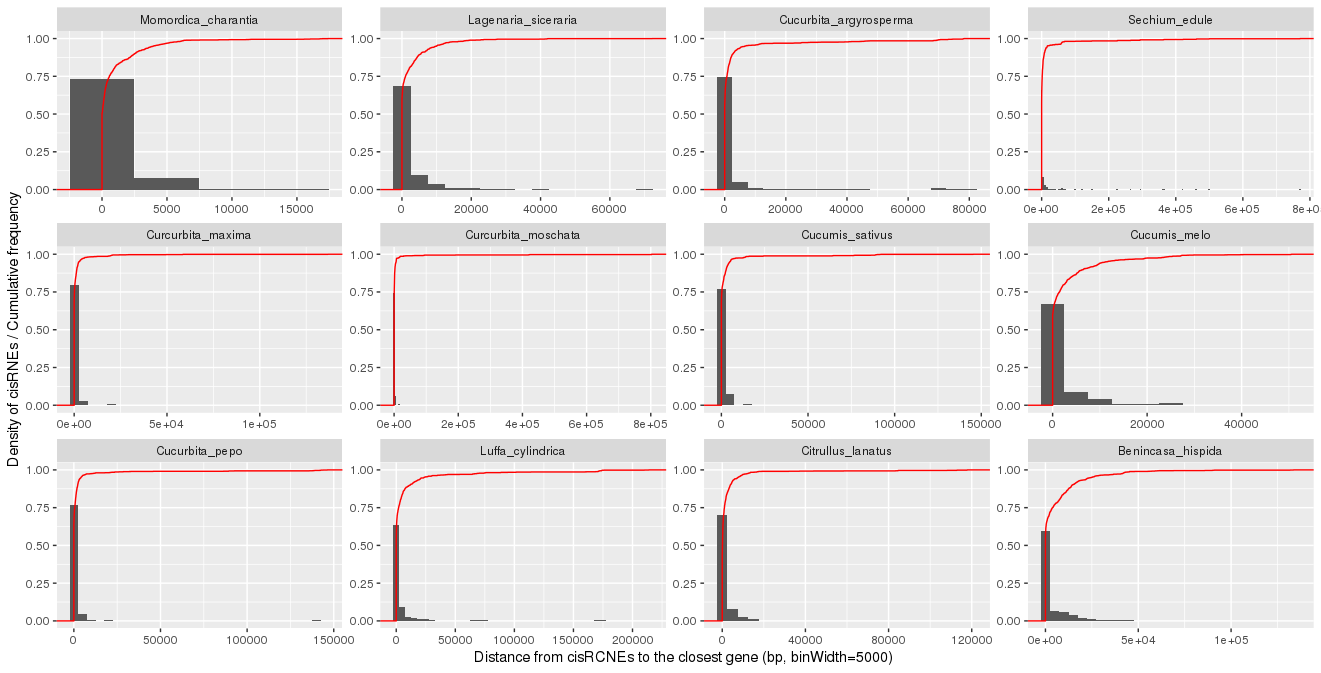


**Fig. S6. One example screenshot of *cis-*RCNEs, collinear segments and nearby genes models from Jbrowse database in cucumber**


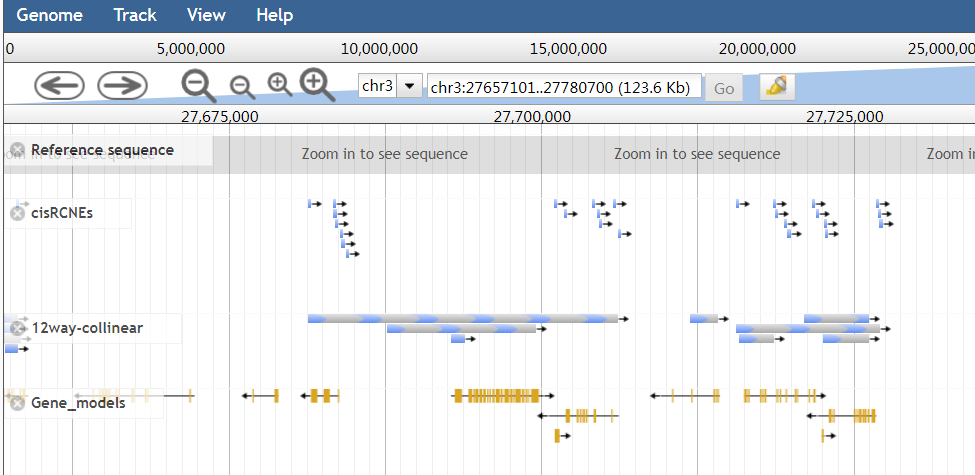


**Fig. S7 Integrative analysis using 1000 times of shuffling DNA elements in cucumber**

H3K27me3 signal according to expression strength as a line plot. Line colors reflect the expression level, while line shapes correspond to averaged H3K27me3 signal across shuffling elements. Line colors reflect the expression level, while line shapes correspond to averaged DNA openness signal across the shuffling elements. Along the X-axis is the location of signal relative to shuffling elements. Fresh fruits 10 days post-anthesis (10DPA) and fresh fruits 40 days post-anthesis (40DPA) were considered for the experiments. All counts with the coding regions were removed from the BAM files to avoid the effect of the coding sequences.


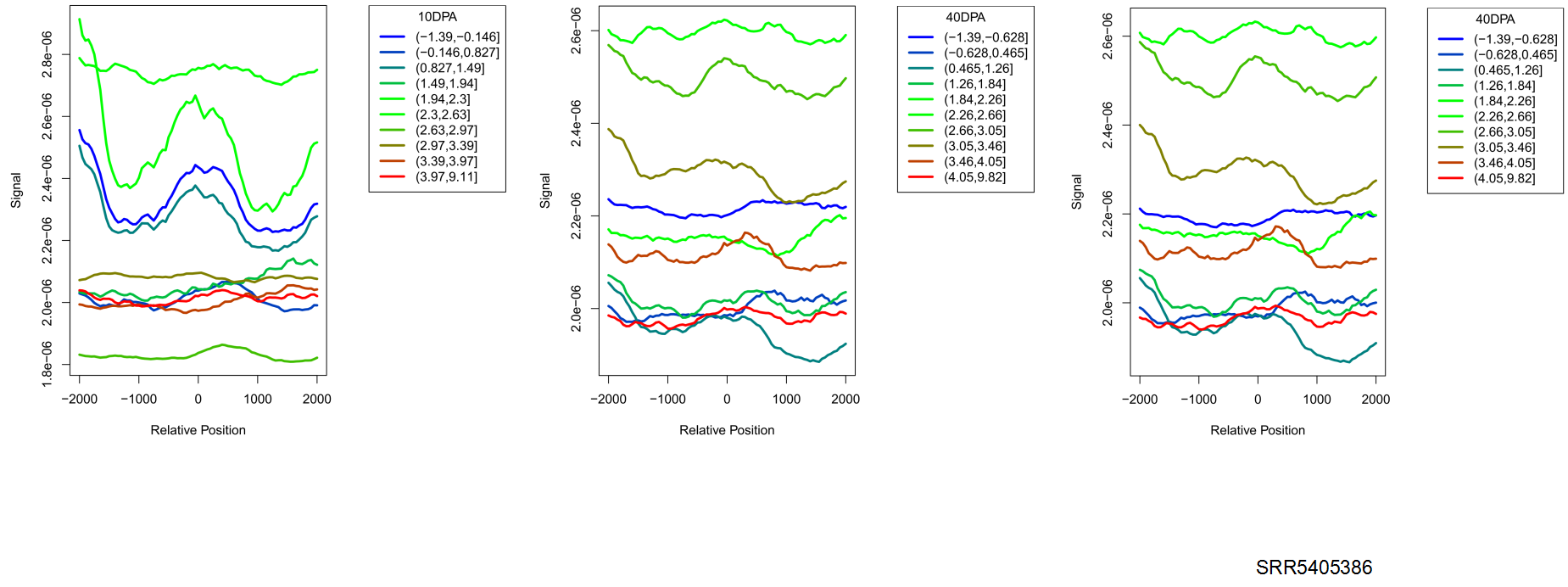


**Fig. S8 A phylogenetic tree of the relationships between species included in the 6-way Solanaceae whole-genome alignmen**t

The neutral tree is based on 4-fold degenerate sites sampled from tomato SL4.0ch01~SL4.0ch12 with branches proportional to the listed scale, with substitutions per site determined by PhyloFit^4^.


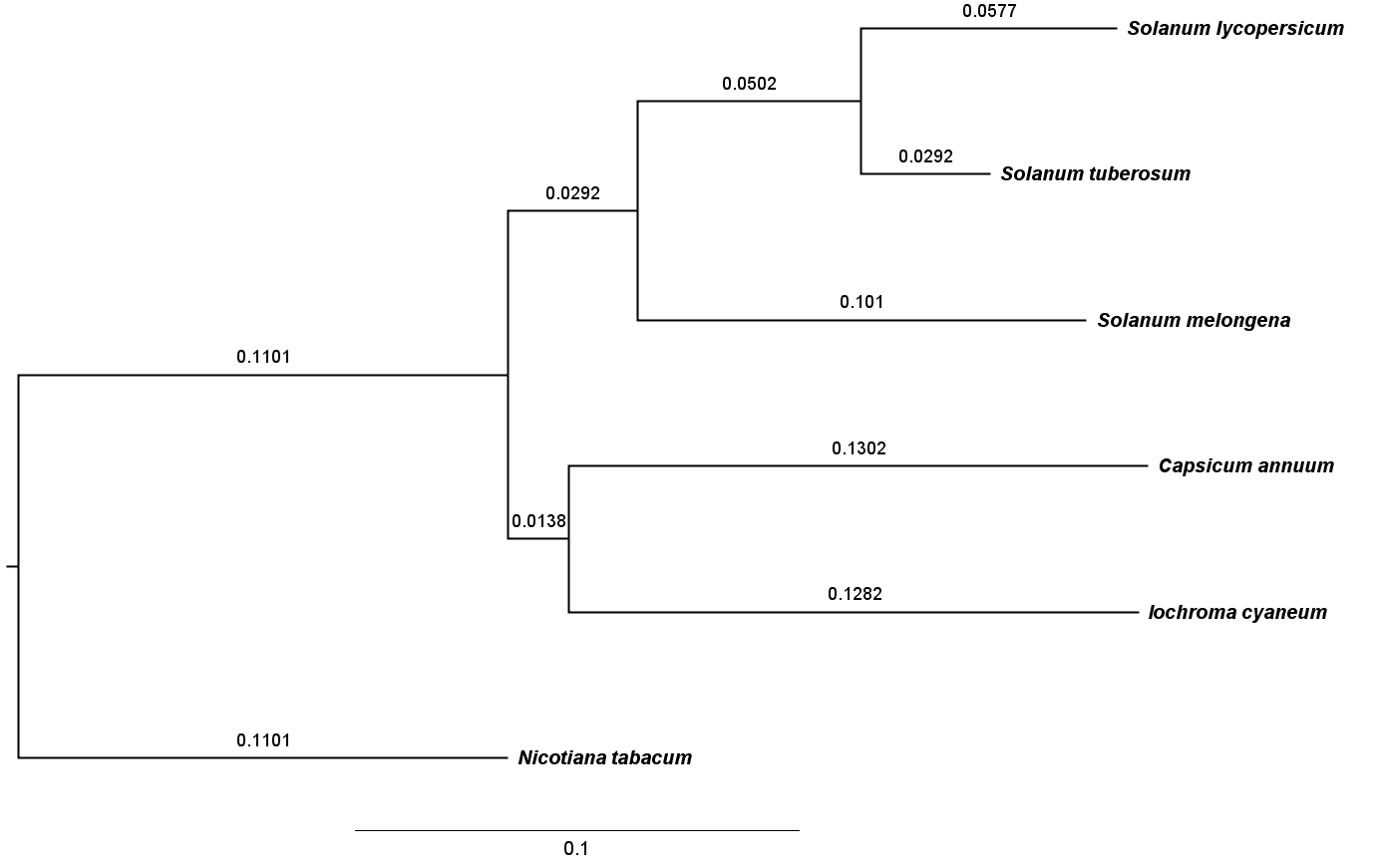


**Fig. S9 Composition of the most-Cons sequences predicted by phastCons in Solanaceae genomes**

The ‘genomeRef’ in the inner cycle and ‘mostCons’ in the outer cycle indicate the composition of the genome and most-Cons sequences predicted from phastCons, respectively. CDS: coding sequences; intergenic: intergenic regions; UTR: untranslated regions.


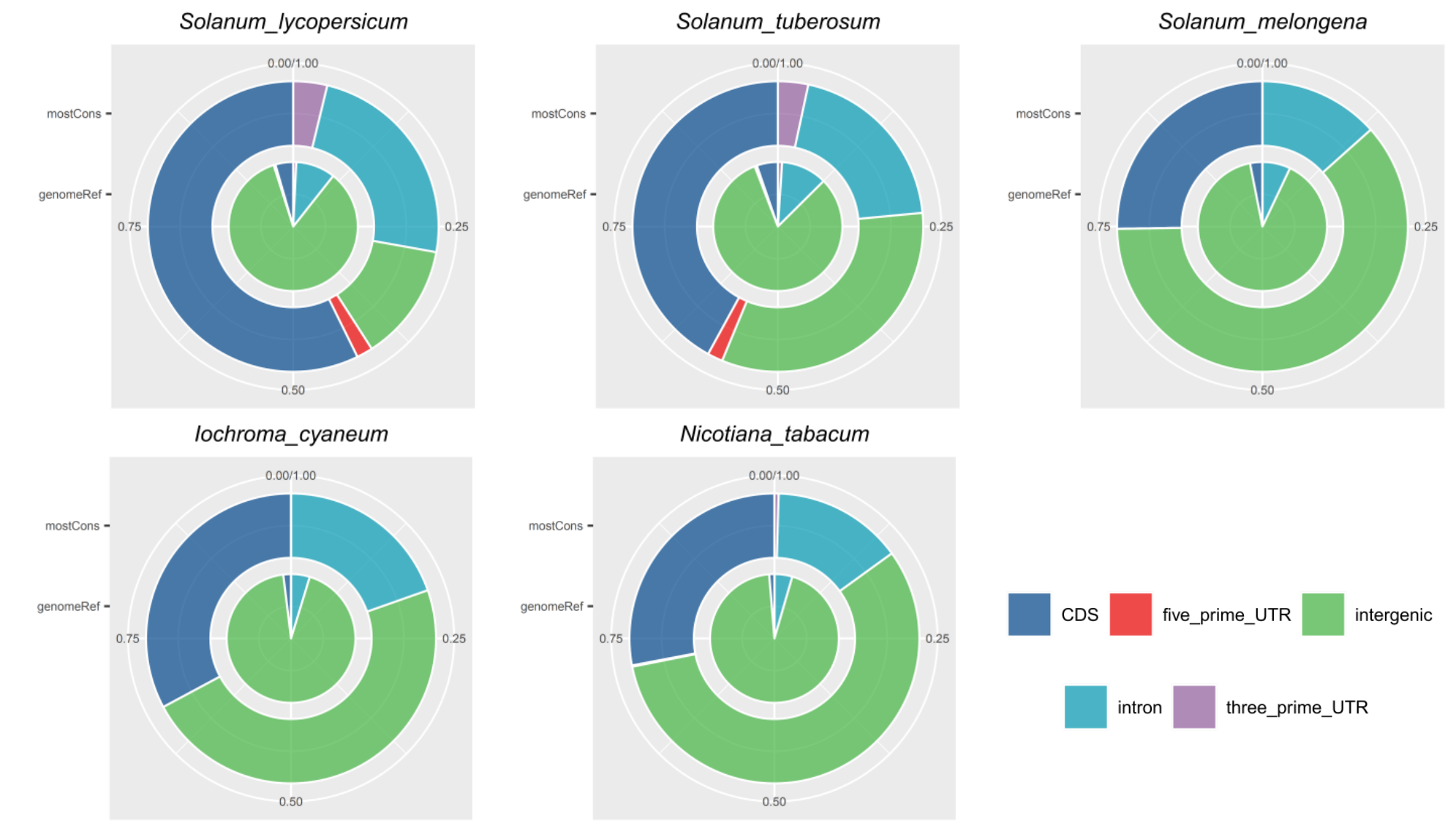


**Fig. S10 Percentage of sites predicted by phyloP for different sets of regions in the tomato genome**

CON: Conservation site. Negative numbers indicate upstream of the gene, and the values in square brackets show the distance (base pairs) from the start codon. Positive numbers indicate downstream of the gene, and the values in square brackets show the distance (bases pairs) from the stop codon. CDS indicates the protein-coding sequences. All flanking regions that overlapped with nearby genes were removed.


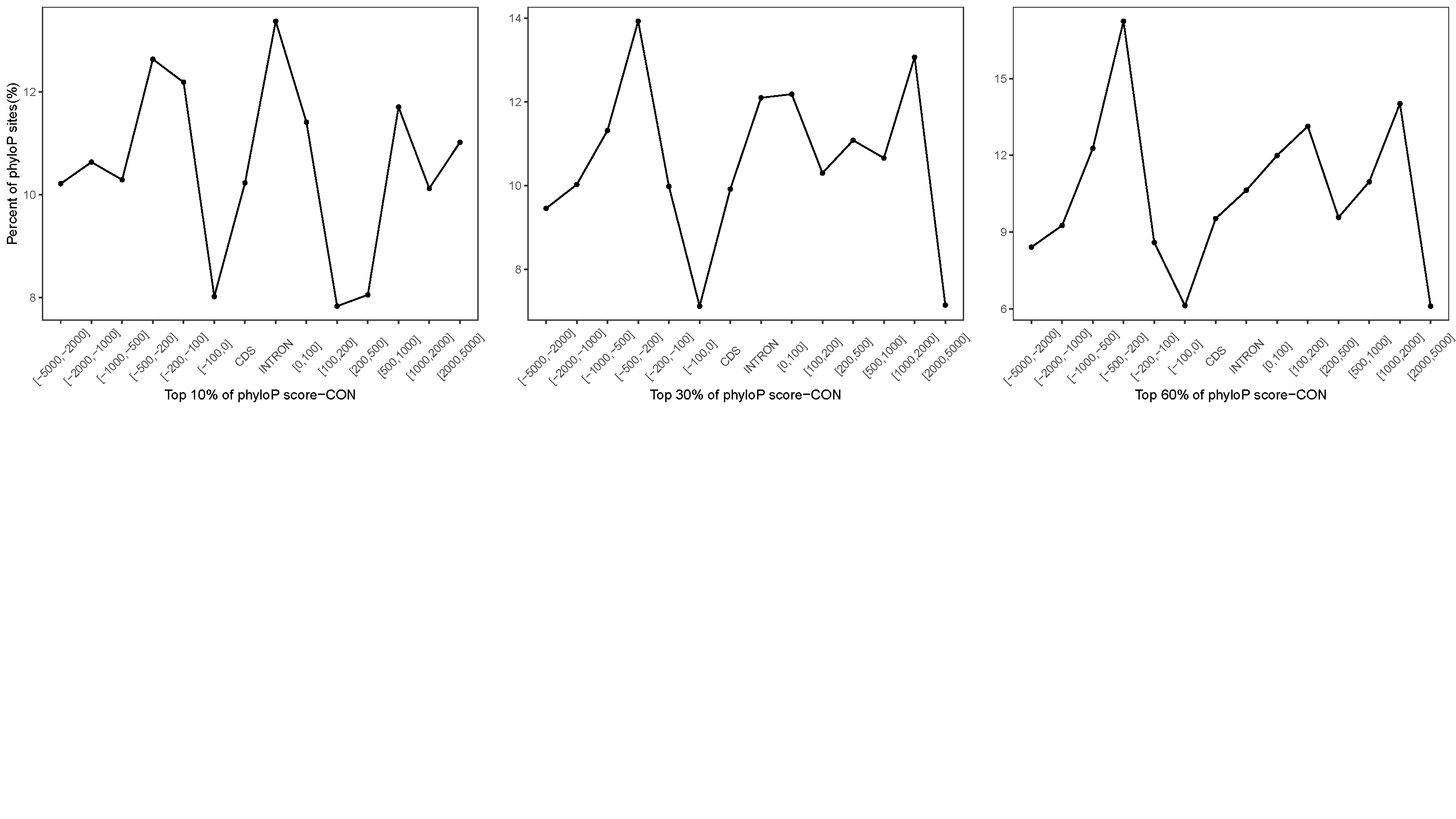


**Fig. S11 Distribution of A+T content and nucleosome occupancy probabilities in the flanking regions and within putative *cis*-RCNEs with the tomato genome as reference**

(a) The distribution of A+T content. Green line: Average A+T content in the tomato genome. Red lines: A+T content inside *cis*-RCNEs (30 bp from the center of each *cis*-RCNE was considered as mentioned in the methodology) acquired through moving window analysis. (b) The distribution of nucleosome occupancy probabilities. The brown line represents the center of each *cis*-RCNE and the center of the random samples. The blue curve shows the nucleosome occupancy probabilities of *cis-*RCNEs, the red curve is a random sample with the same A+T content as *cis*-RCNEs, and the green curve is a random sample without a specific A+T content preference.


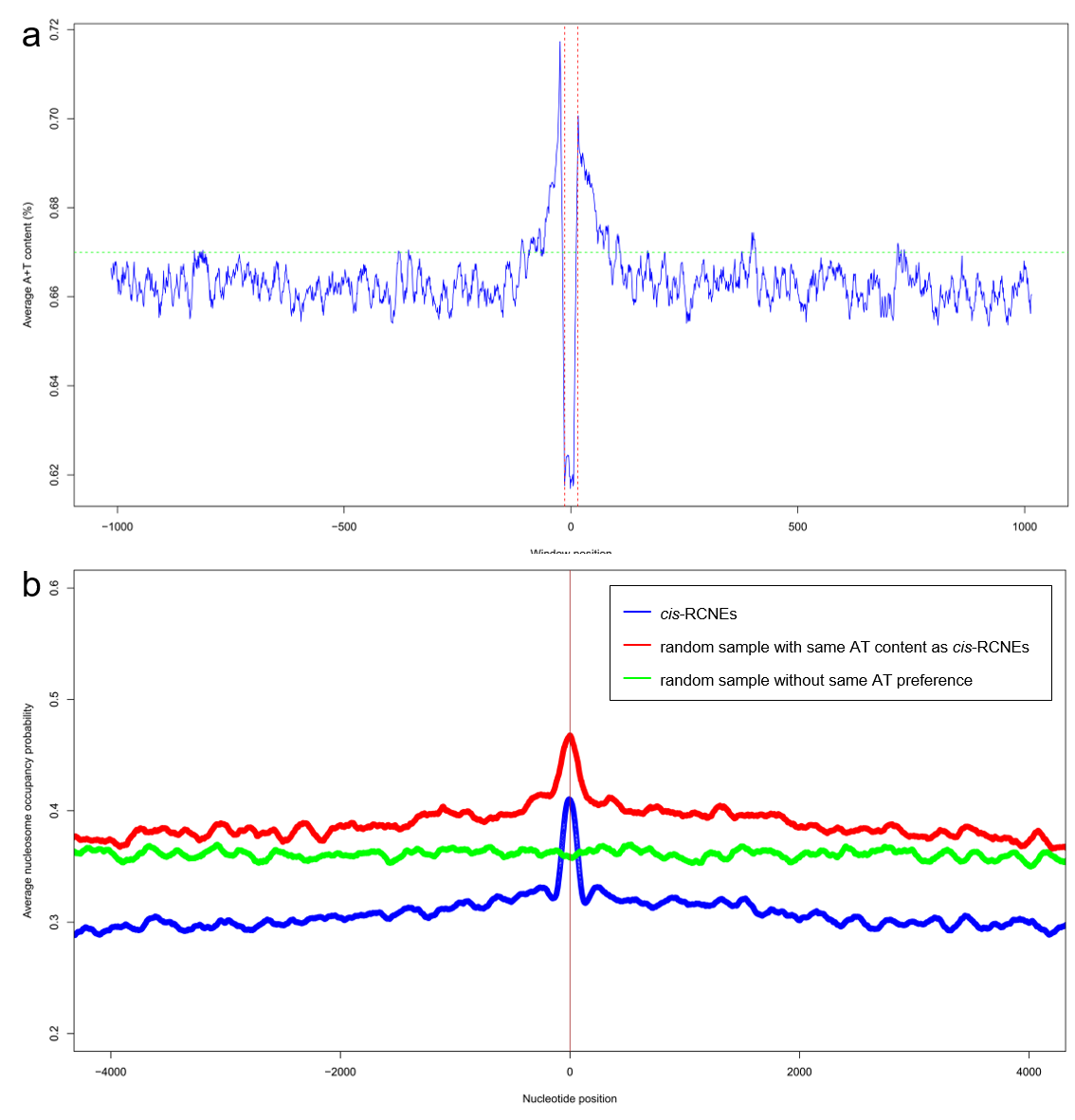


**Fig. S12 The H3K27me3 signal near the center of putative *cis*-RCNEs in regulating fruit ripening-regulated genes of tomato**

(a) All putative *cis*-RCNEs in three replications of wide type fruit; (b) Subset of *cis*-RCNEs whose flanking overlapped with one up-regulated gene at least in SlJMJ6-overexpressed fruit compared with WT in three replications of SlJMJ6-overexpressed fruit. ChIP-seq and RNA-seq raw datasets were from ^5^, and SlJMJ6 encodes a histone lysine demethylase that specifically demethylates H3K27 methylation. The line plot indicates the H3K27me3 signal according, whereas line shapes correspond to the averaged H3K27me3 signals across the putative *cis*-RCNEs. Along the x-axis is the location of the signal relative to the *cis*-RCNEs. Zero in the x-lab indicates the center of putative *cis*-RCNEs. All counts with the coding regions were removed from the BAM files to avoid the effect of the coding sequences on the *cis-*RCNEs.


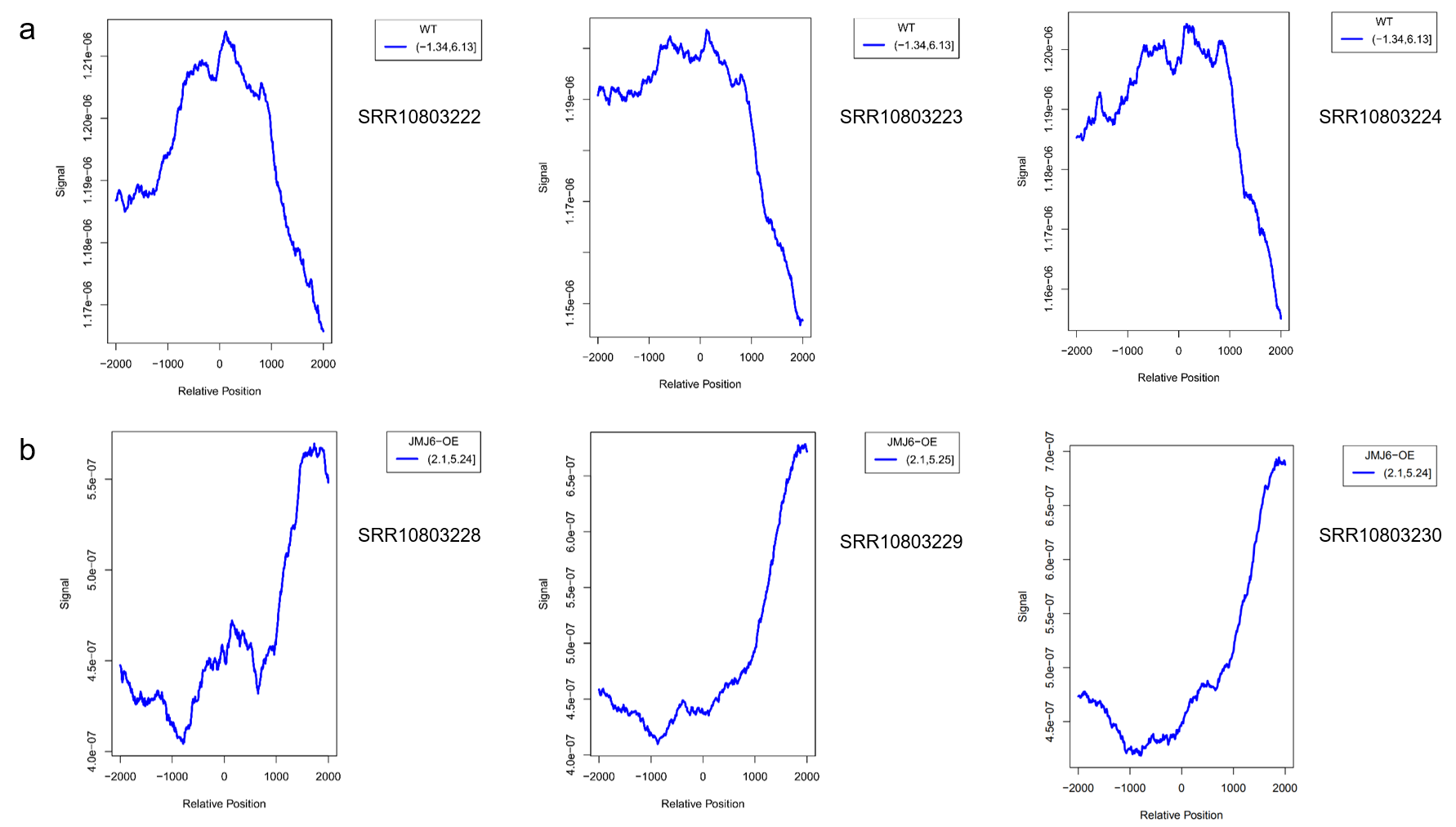


**Table S1-S11 were uploaded as Supplementary Tables file.**

**References:**

1. Kozlov, A.M., Darriba, D., Flouri, T., Morel, B. & Stamatakis, A. RAxML-NG: a fast, scalable and user-friendly tool for maximum likelihood phylogenetic inference. *Bioinformatics* **35**, 4453-4455 (2019).

2. Emms, D.M. & Kelly, S. OrthoFinder: phylogenetic orthology inference for comparative genomics. *Genome Biol* **20**, 238 (2019).

3. Kumar, S., Stecher, G. & Tamura, K. MEGA7: Molecular Evolutionary Genetics Analysis Version 7.0 for Bigger Datasets. *Mol Biol Evol* **33**, 1870-4 (2016).

4. Siepel, A. et al. Evolutionarily conserved elements in vertebrate, insect, worm, and yeast genomes. *Genome Res* **15**, 1034-50 (2005).

5. Li, Z. et al. Histone demethylase SlJMJ6 promotes fruit ripening by removing H3K27 methylation of ripening-related genes in tomato. *New Phytol* **227**, 1138-1156 (2020).
