# Supplementary figures and images for: Identification of clade-wide putative cis-regulatory elements from conserved non-coding sequences in Cucurbitaceae genomes
Source: Hortic Res. 2023 Feb 28;10(4):uhad038. doi: 10.1093/hr/uhad038 (PMC10548412; doi:10.1093/hr/uhad038)

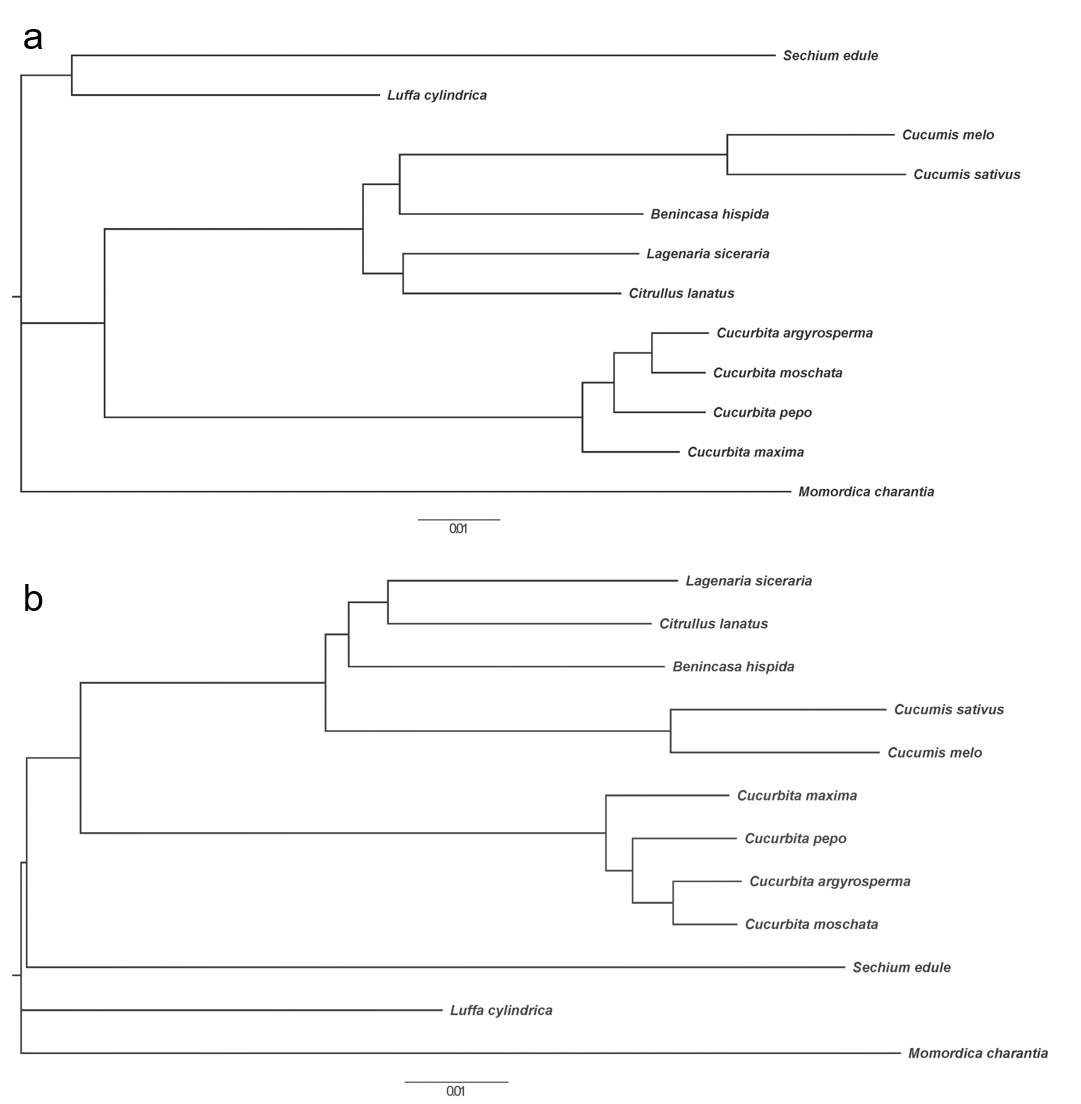

Supplement: Web_Material_uhad038 [file web_material_uhad038.zip › Supp_Fig_S1.tif]

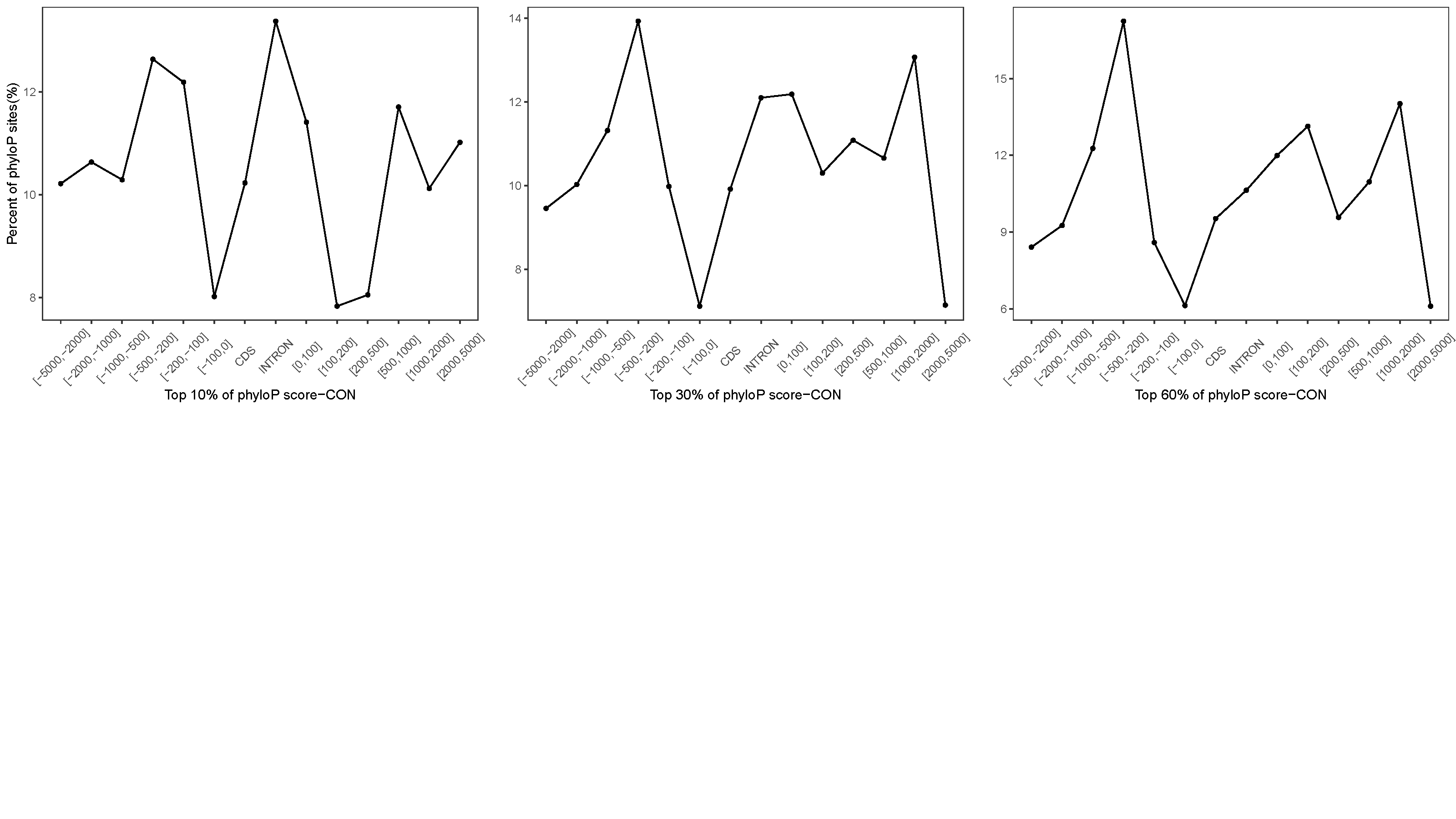

Supplement: Web_Material_uhad038 [file web_material_uhad038.zip › Supp_Fig_S10.tiff]

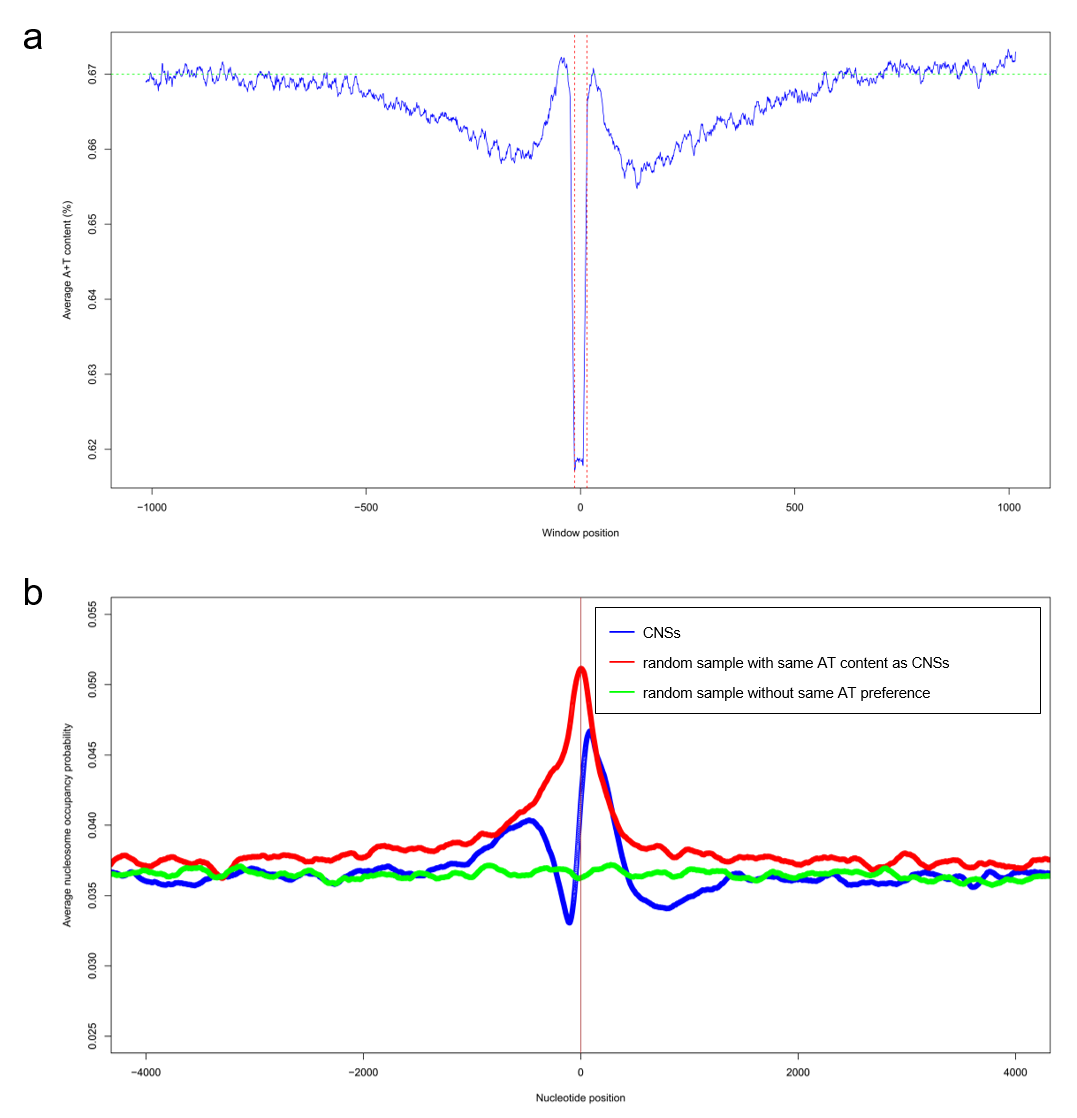

Supplement: Web_Material_uhad038 [file web_material_uhad038.zip › Supp_Fig_S11.tif]

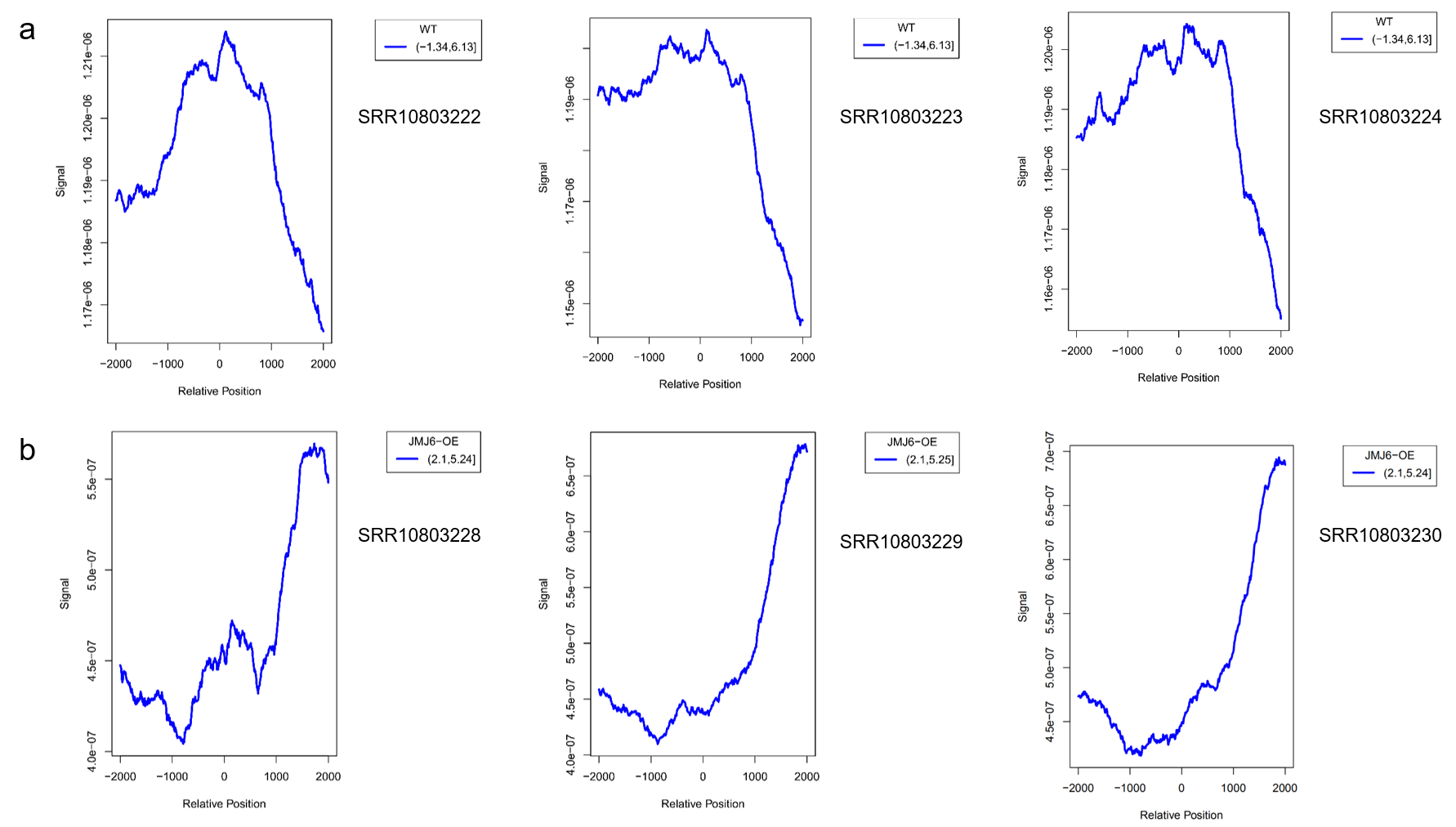

Supplement: Web_Material_uhad038 [file web_material_uhad038.zip › Supp_Fig_S12.tif]

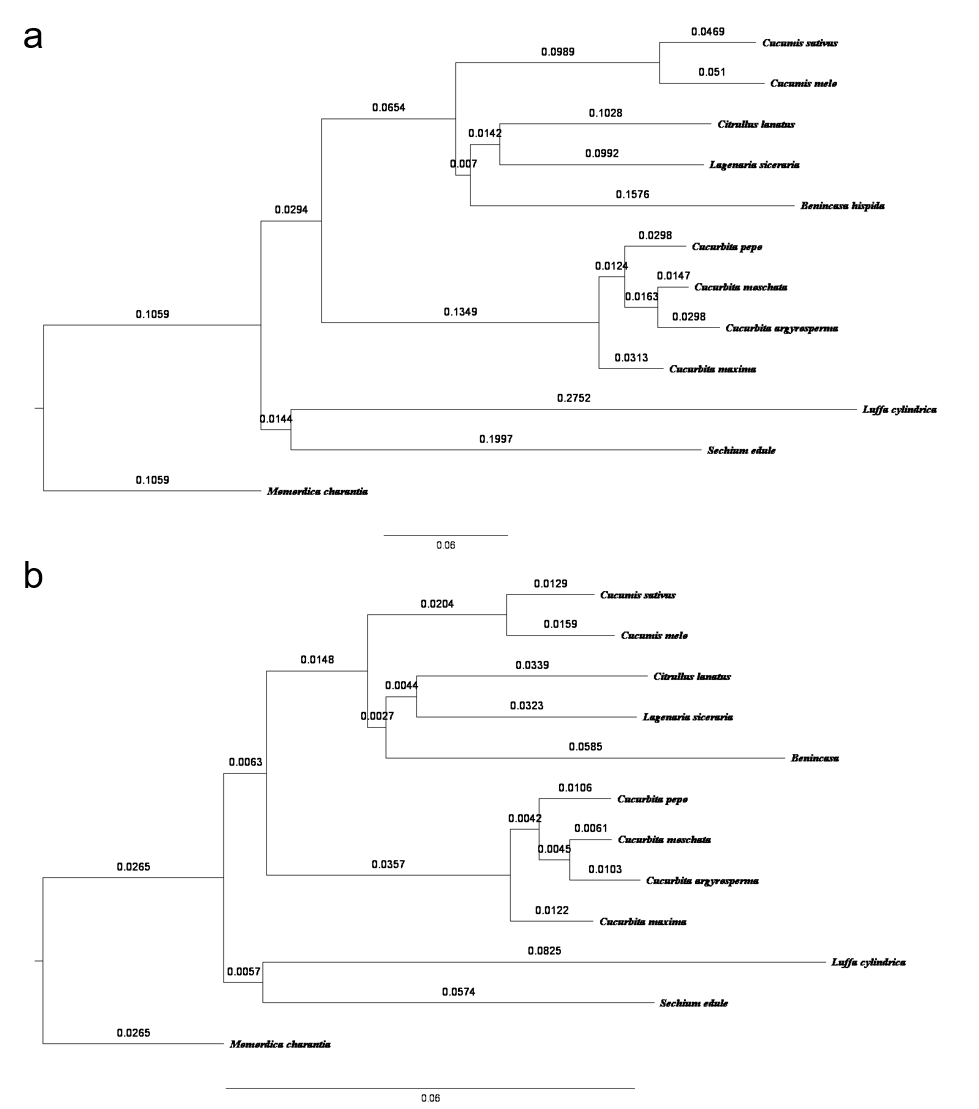

Supplement: Web_Material_uhad038 [file web_material_uhad038.zip › Supp_Fig_S2.tiff]

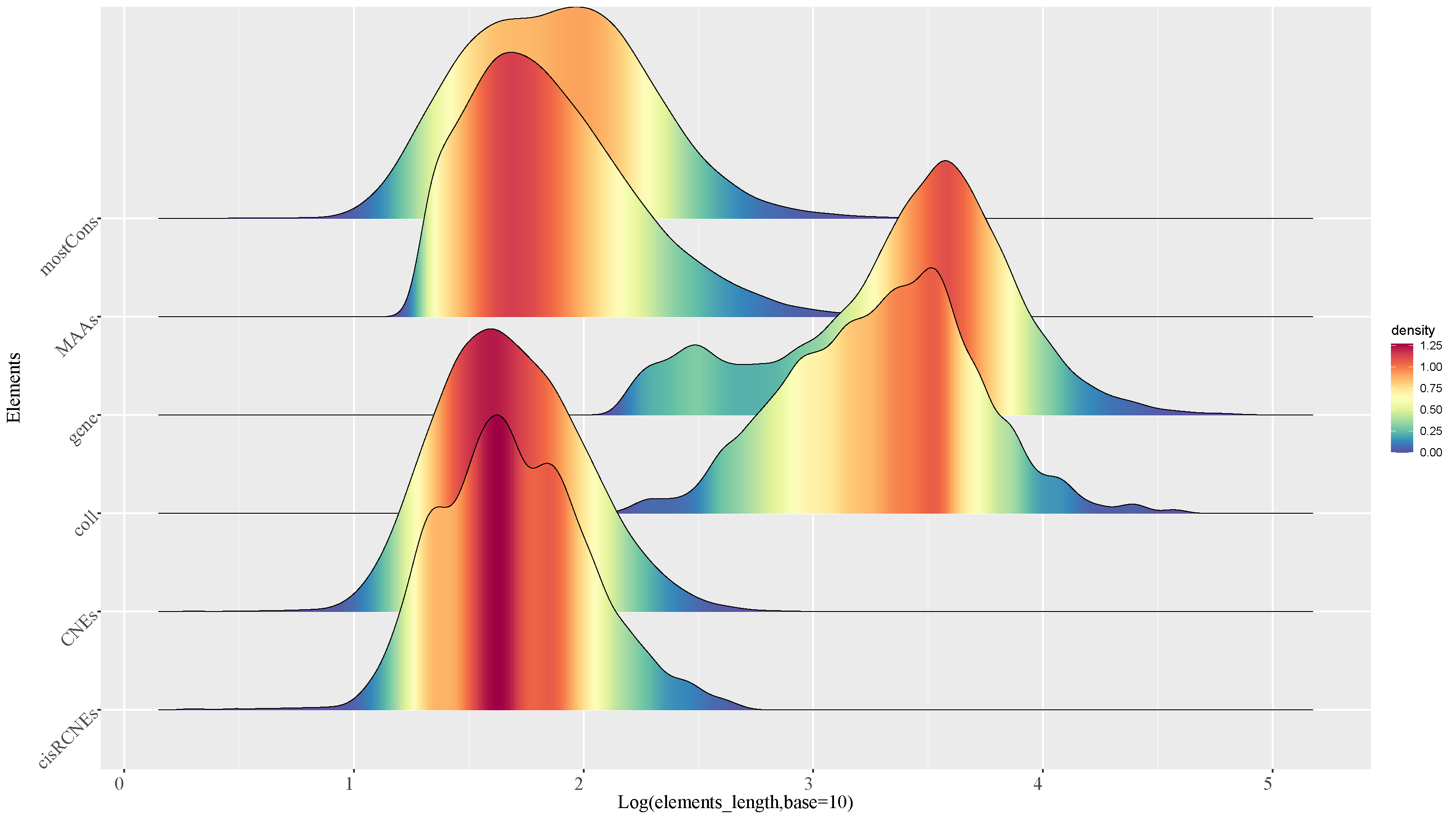

Supplement: Web_Material_uhad038 [file web_material_uhad038.zip › Supp_Fig_S3.tiff]

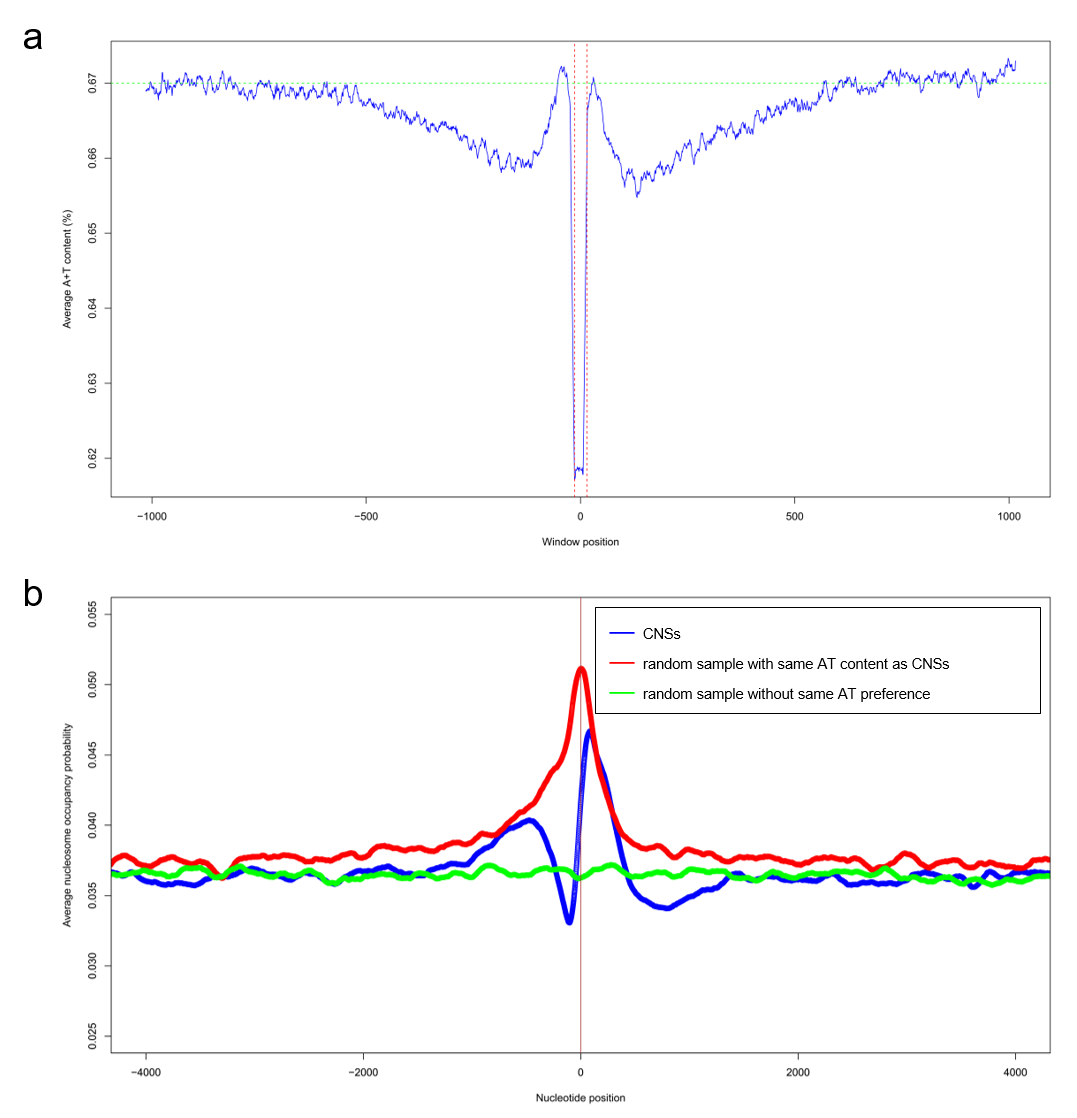

Supplement: Web_Material_uhad038 [file web_material_uhad038.zip › Supp_Fig_S4.tif]

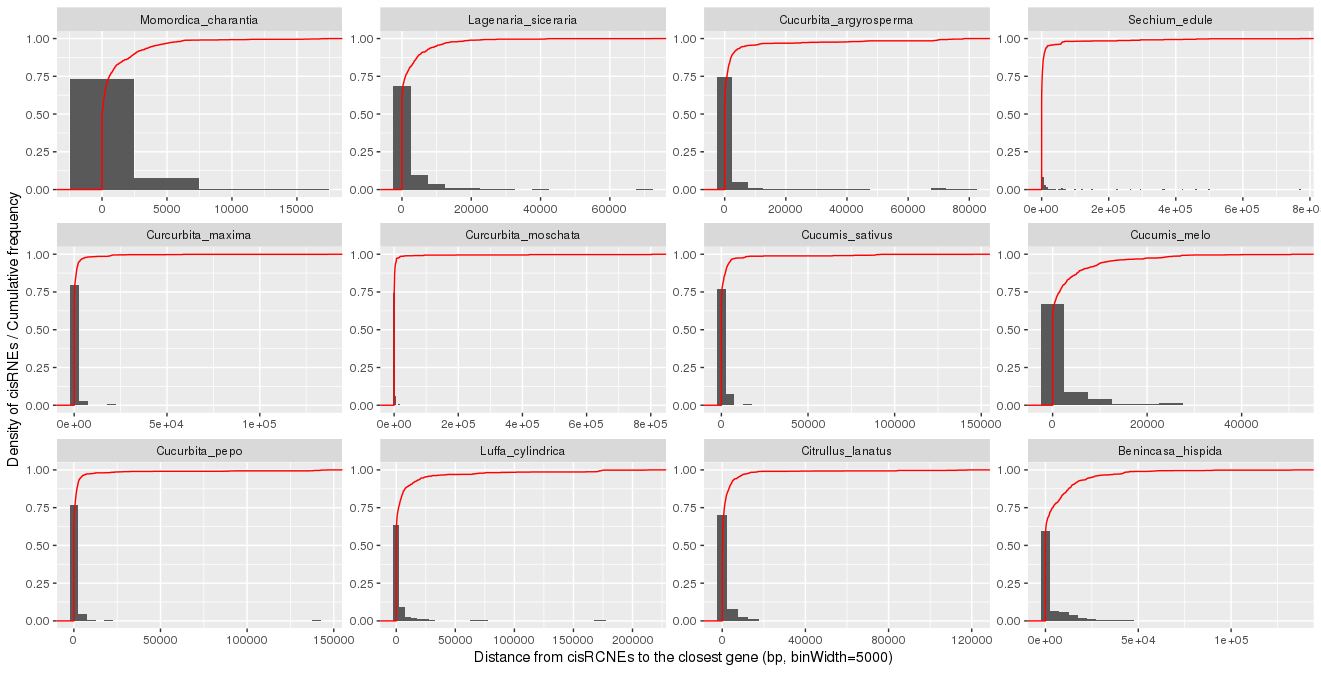

Supplement: Web_Material_uhad038 [file web_material_uhad038.zip › Supp_Fig_S5.tiff]

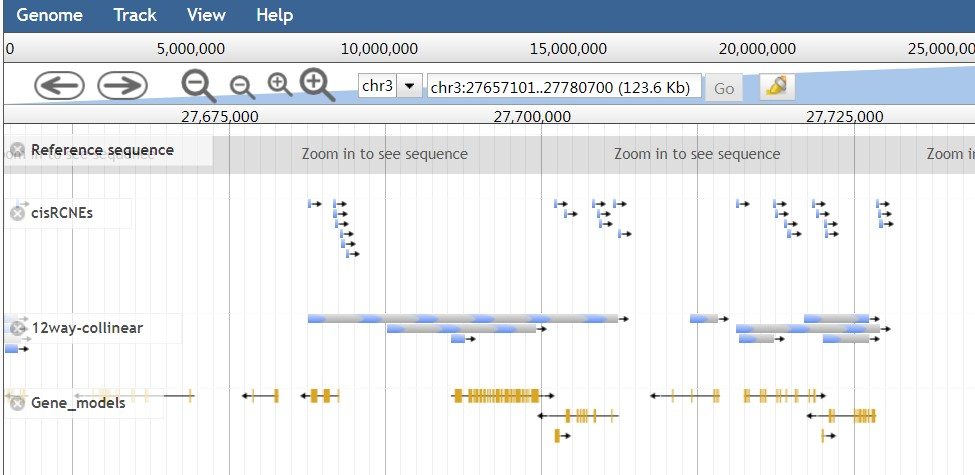

Supplement: Web_Material_uhad038 [file web_material_uhad038.zip › Supp_Fig_S6.tiff]

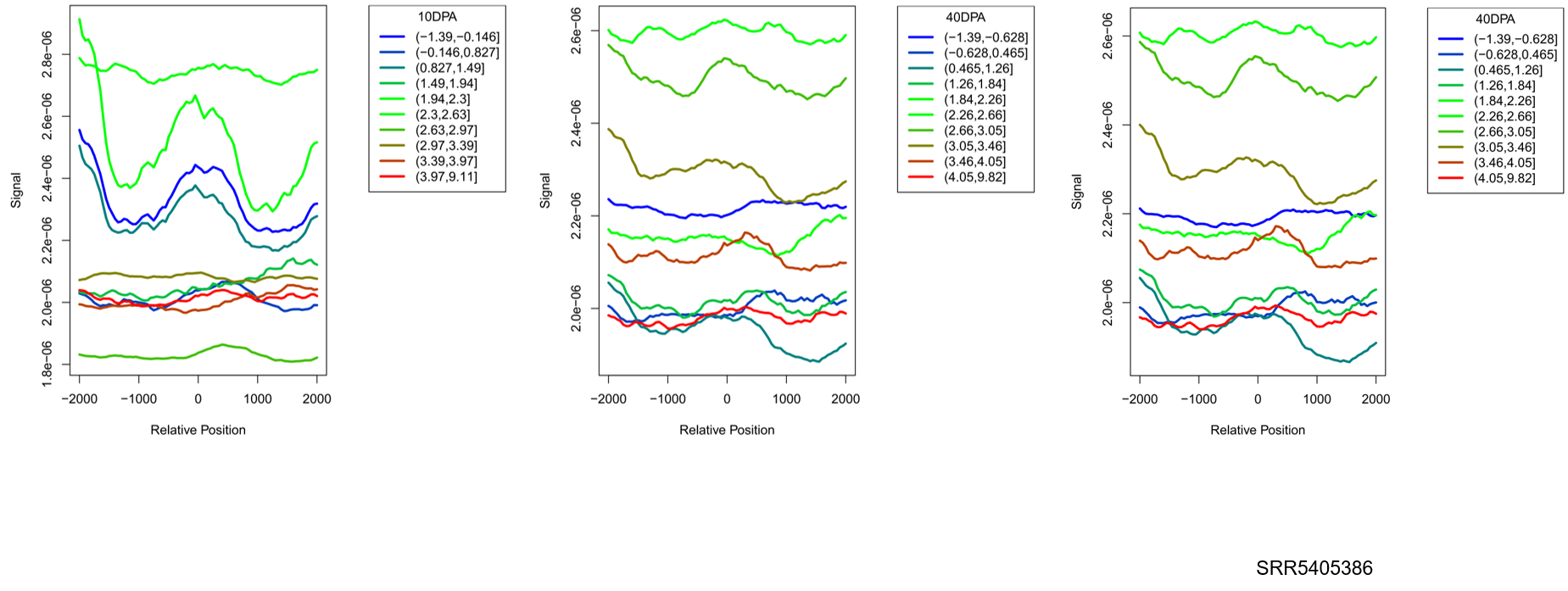

Supplement: Web_Material_uhad038 [file web_material_uhad038.zip › Supp_Fig_S7.tiff]

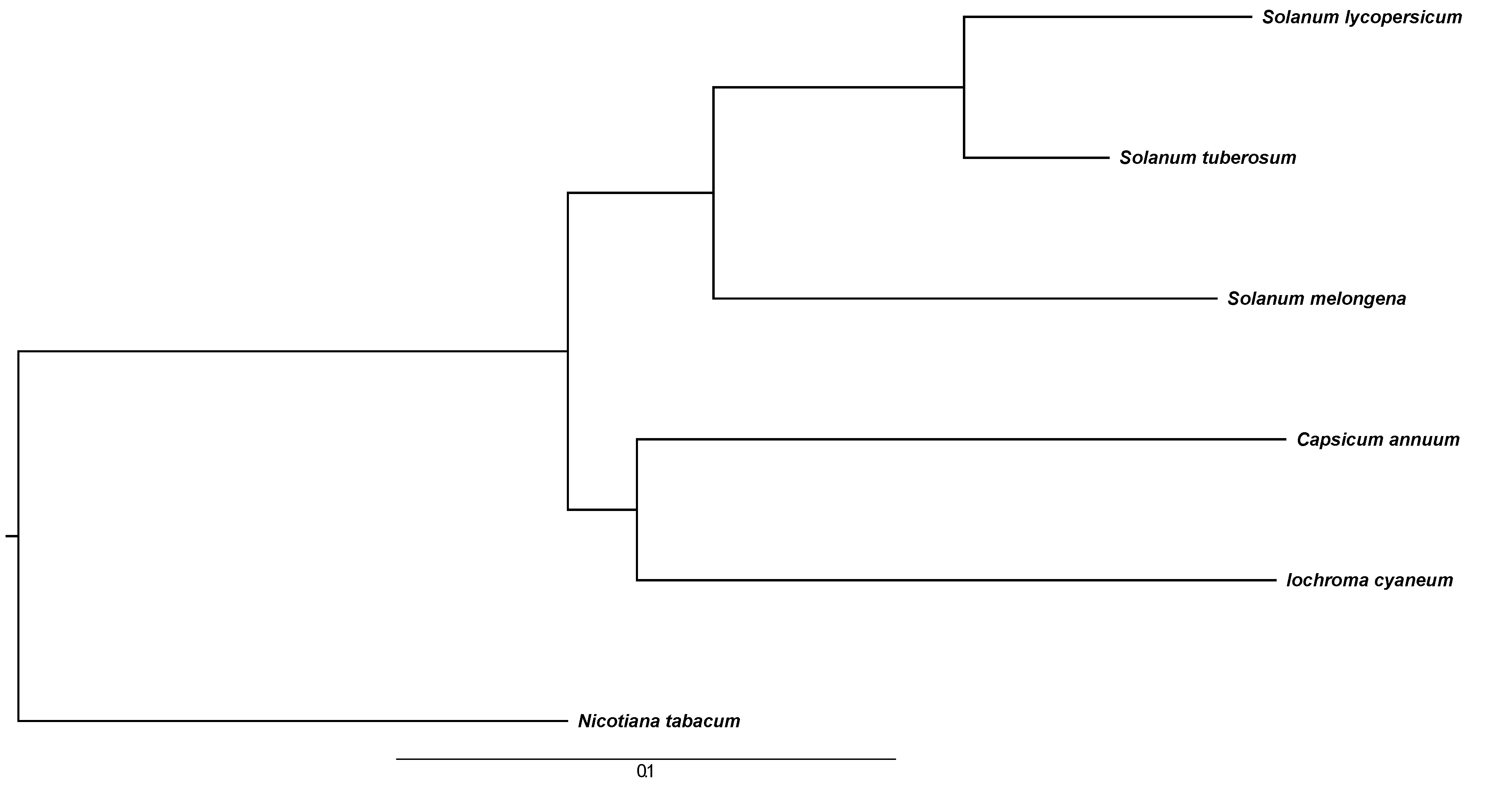

Supplement: Web_Material_uhad038 [file web_material_uhad038.zip › Supp_Fig_S8.tiff]

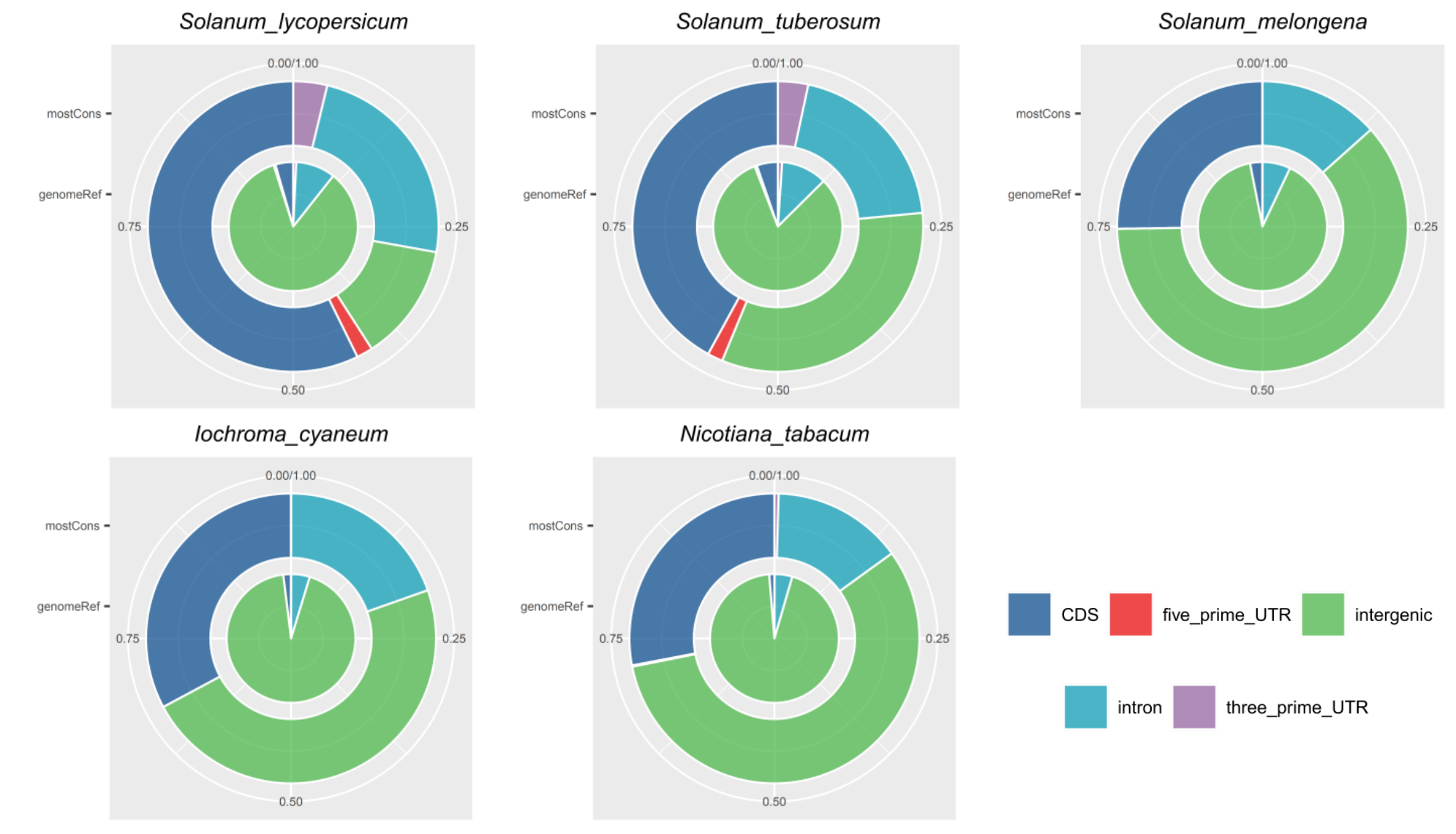

Supplement: Web_Material_uhad038 [file web_material_uhad038.zip › Supp_Fig_S9.tiff]
